# Comparative Analysis of Gut Microbiota Patterns in Irritable Bowel Syndrome, Anxiety, and Autoimmune Disorders

**DOI:** 10.3390/biomedicines14051005

**Published:** 2026-04-28

**Authors:** Adelin-Rareș Candrea, Laura Ioana Gavrilaș, Andrei Mocan, Adriana Rusu, Gianina Crișan

**Affiliations:** 1 Department of Pharmaceutical Botany, Faculty of Pharmacy, “Iuliu Hațieganu” University of Medicine and Pharmacy, 23 Gheorghe Marinescu Street, 400337 Cluj-Napoca, Romania; adelin.rare.candrea@elearn.umfcluj.ro (A.-R.C.);; 2 Department of Development, Innovation and Research, Romanian Dietitians Association, 17A, Janos Zsigmond Street, 400347 Cluj-Napoca, Romania; 3 2nd Department, Faculty of Nursing and Health Sciences, “Iuliu Hațieganu” University of Medicine and Pharmacy, 23 Gheorghe Marinescu Street, 400337 Cluj-Napoca, Romania; 4 Diabetes Center, Emergency Clinical County Hospital Cluj, 400006 Cluj-Napoca, Romania

**Keywords:** gut microbiota, irritable bowel syndrome, anxiety, autoimmune disorders

## Abstract

**Background/Objectives**: The gut microbiota has been increasingly studied in the context of gastrointestinal, neuropsychiatric, and immune-mediated disorders. However, comparative data across these conditions within the same population remain limited. This observational study aimed to perform a comparative analysis of gut microbiota composition across individuals with irritable bowel syndrome (IBS), anxiety disorders (AG), and autoimmune diseases (AI) and healthy controls (HC) from a Romanian cohort, aiming to identify population-specific microbial patterns. **Methods**: This study included 59 Romanian adults categorized into IBS (*n* = 27), AG (*n* = 13), AI (*n* = 11), and HC (*n* = 8) groups. Gut microbiota profiles were analyzed using 16S rRNA gene sequencing (NGS), and fecal short-chain fatty acids were quantified by headspace gas chromatography–mass spectrometry. **Results**: At the phylum level, Actinobacteria showed significantly higher relative abundance in HC compared to both the AI (adjusted *p* = 0.028) and the AG (adjusted *p* = 0.023) groups, with the AG–HC difference confirmed by LinDA analysis (FDR-adjusted *p* = 0.049). At the genus level, *Bifidobacterium* spp. was significantly lower in the AG compared to HC (adjusted *p* = 0.036), while *Bacteroides* spp. showed higher abundance in the AG compared to AI (adjusted *p* = 0.013), a finding supported by Kruskal–Wallis analysis. Lactate production was significantly lower in both the AI (*p* = 0.014) and AG (*p* = 0.038) compared to HC. The Simpson index revealed significantly lower microbial evenness in the AG compared to IBS and AI (FDR-adjusted *p* = 0.042). Beta diversity analysis identified significant differences in community composition between groups (PERMANOVA: Pseudo-F = 2.395, R^2^ = 0.116, *p* = 0.039), with the most distinct separation between AI and AG (FDR-adjusted *p* = 0.048). Spearman correlation analysis revealed strong positive associations between *Bifidobacterium* and lactate, *Bacteroides* and the acetate/propionate ratio, and *Actinobacteria* and lactate (all FDR-adjusted *p* < 0.001). **Conclusions**: The present study provides exploratory evidence of disease-associated microbial and functional patterns in a Romanian cohort, with reduced Actinobacteria in the anxiety group as the most robust observation, suggesting a potential role of the gut microbiota in gut–brain–immune interactions and contributing preliminary population-specific data from an underrepresented Eastern European population.

## 1. Introduction

The human gut microbiota represents a dense and metabolically active microbial ecosystem that varies across individuals and populations and is increasingly investigated in relation to gastrointestinal [1], psychological [2], and immune-mediated disorders [3]. In clinical research, stool-based 16S rRNA sequencing and metagenomic approaches have repeatedly shown that patients with chronic conditions may present alterations in microbial diversity and community structure compared with healthy controls (HC); however, the magnitude and direction of these differences often vary between cohorts, diagnostic subtypes, and geographic settings [4].

In irritable bowel syndrome (IBS), systematic reviews and meta-analyses of human case–control studies indicate that dysbiosis is frequently reported, yet results remain heterogeneous and sometimes contradictory across studies, reflecting differences in diagnostic criteria, IBS subtype distribution, diet, medication exposure, and sequencing methods [4,5]. Irritable bowel syndrome affects approximately 5% of the population and is a disorder of gut–brain interaction, characterized by recurrent abdominal pain associated with changes in bowel habits, leading to a significant impairment in quality of life [6]. Clinical microbiome studies in IBS show that some patients have different fecal microbial profiles compared with healthy individuals, including changes in overall microbial composition and, in certain groups, lower alpha diversity. However, no consistent microbial pattern has been identified yet across all populations [4,5]. This variability suggests that IBS is not a single condition but rather a group of different endophenotypes. In these subgroups, microbiome alterations may be linked to distinct biological pathways, including gut barrier dysfunction, immune activation, and changes in gut–brain communication, potentially influenced by dietary habits and environmental exposures [7].

In addition to gastrointestinal disorders, differences in the gut microbiota are linked to anxiety-related traits and anxiety disorders. Systematic reviews focusing on depression and anxiety report compositional differences in the gut microbiota between affected individuals and HC, while also emphasizing substantial variability between studies and the influence of confounding factors such as psychotropic medication use, dietary habits, and coexisting physical conditions [8,9]. Preclinical studies provide evidence for a causal role of the gut microbiota in anxiety-related behaviors. In a mouse model, innate high- and low-anxiety phenotypes were associated with distinct gut microbial profiles, with low-anxiety mice showing increased relative abundances of taxa such as the genera *Lactobacillus* and *Parabacteroides*, and the phylum Campylobacterota. Microbiota depletion via antibiotic treatment induced anxiolytic-like effects, while fecal microbiota transplantation from high-anxiety donors transferred anxiety-like behaviors to recipient mice, accompanied by altered neural activity in key anxiety-related brain regions [10]. In line with preclinical findings, human Mendelian randomization analyses indicate that specific gut microbial taxa may exert protective or risk effects in anxiety, with Actinobacteria, Bifidobacteriales, and *Parasutterella* showing protective associations, and Oxalobacteraceae and *Alistipes* being linked to higher anxiety risk [11]. Moreover, a recent disorder-specific clinical study has reported altered gut microbiota diversity and composition in panic disorder, compared with HC [12]. Collectively, these findings suggest that gut microbiota alterations may contribute to anxiety-related phenotypes through microbiota–gut–brain pathways, influencing stress responsivity and symptom expression in a subset of individuals.

Microbiota alterations are also clinically relevant in immune-mediated and autoimmune diseases, where human studies indicate associations between dysbiosis and immune activity, disease phenotype, or disease stage [13]. Certain microbial taxa, including *Lactobacillus* and *Bifidobacterium*, have been suggested to interact with thyroid autoimmunity through molecular mimicry mechanisms, due to structural similarities between bacterial proteins and thyroid-specific antigens. Dietary patterns and drug use may contribute to gut dysbiosis, potentially disrupting the microbial balance and indirectly affecting thyroid function [14]. For instance, a clinical study reported differences in gut microbiota composition between healthy individuals and patients with Hashimoto’s thyroiditis, with increased abundances of *Akkermansia*, *Bifidobacterium*, and Lachnospiraceae in patients, while taxa such as *Lachnoclostridium*, *Bilophila*, and *Klebsiella* were more abundant in HC. Certain bacteria were further associated with thyroid hormone levels, suggesting a potential link between gut microbiota and thyroid autoimmunity [15]. In line with findings from autoimmune thyroid disease, preclinical and clinical evidence also indicates altered gut microbiota composition in patients with psoriasis compared with healthy individuals, reinforcing the notion that gut dysbiosis may contribute to immune-mediated pathologies beyond the thyroid [16]. In addition to autoimmune thyroid disease and psoriasis, autoimmune gastritis is increasingly recognized as an immune-mediated disorder of the gastric mucosa, characterized by chronic inflammation and progressive loss of acid-secreting cells [17]. While the specific clinical evidence directly linking gastric and gut microbiota alterations to autoimmune gastritis is still emerging, studies in hypochlorhydric and atrophic gastritis states, including autoimmune forms, indicate shifts in microbial diversity and composition compared with HC, supporting the concept that immune-driven disruptions of mucosal barriers and local inflammatory signals may influence the gastric and potentially intestinal microbiota, in line with patterns observed in other organ-specific autoimmune diseases [17,18].

Despite increasing interest in the role of the gut microbiota in IBS, anxiety disorders, and autoimmune diseases, substantial knowledge gaps persist, particularly regarding comparative microbiota profiles across these conditions within the same population. Moreover, most human microbiome studies to date have been conducted in Western European, North American, or East Asian cohorts, with limited representation of Eastern European populations, restricting generalizability and the identification of population-specific microbial patterns [19,20]. These limitations underscore the need for studies assessing gut microbiota composition in diverse populations and across overlapping functional and immune-mediated conditions. Therefore, the present observational study aimed to conduct a comparative analysis of gut microbiota patterns in a Romanian population with IBS, anxiety, and autoimmune disorders. The study further aimed to compare gut microbiota profiles across these groups, contributing population-specific insights into microbiome-related patterns across gastrointestinal, neuropsychiatric, and immune disorders.

## 2. Materials and Methods

### 2.1. Study Design and Enrolled Participants

This study was designed as retrospective, observational, and comparative, analyzing previously collected data. The main objective of this study was to characterize the gut microbiota in a Romanian adult cohort across four distinct clinical categories: healthy individuals (controls), patients with irritable bowel syndrome, patients with anxiety disorders, and patients with autoimmune diseases. A total of 59 subjects were selected from the clinical records of a private nutrition and dietetics clinic in Cluj-Napoca, Romania. The study included patients who attended the private nutrition and dietetics clinic during the period from May 2021 to December 2025. Participant selection was conducted based on predefined inclusion and exclusion criteria. The study population consisted of Romanian adults (≥18 years) whose medical data were available in clinical records and who had previously undergone gut microbiota analysis prior to the initiation of the nutritional intervention. Eligible participants were categorized into four groups.

The HC group consisted of individuals without a diagnosis of chronic gastrointestinal, psychiatric, or autoimmune disease and with a body mass index within the normal range. The irritable bowel syndrome group included patients diagnosed by a medical specialist in accordance with the Rome IV criteria. The anxiety disorders group (AG) comprised patients with a diagnosis of anxiety disorder confirmed by a medical specialist. The autoimmune diseases group (AI) included patients diagnosed by a physician with rheumatoid arthritis, psoriasis, psoriatic arthritis, autoimmune gastritis, or autoimmune thyroiditis. Inclusion criteria included age ≥ 18 years, availability of complete medical records, a diagnosis confirmed by a medical specialist for the pathological groups, and the availability of previously obtained gut microbiota analysis, serum, and anthropometric data. Exclusion criteria were: presence of active malignant disease or chronic conditions other than those targeted by the study; pregnancy or breastfeeding at the time data were recorded; use of antibiotics, probiotics, or anti-inflammatory medications prior to the gut microbiota analysis.

### 2.2. Data Collection and Gut Microbiota Analysis

To minimize temporal variability, only participants for whom blood tests and stool samples for gut microbiota analysis were collected within the same week were included. Gut microbiota profiling was performed by a certified external laboratory (Bioclinica, Romania, in partnership with Ganzimmun Diagnostics, Germany, an ISO/IEC 17025-accredited commercial laboratory) using 16S rRNA gene amplicon sequencing [21]. The hypervariable V3–V4 regions of the 16S rRNA gene were amplified using primers 341F and 805R. Sequencing was performed on an Illumina MiSeq i100 Plus (San Diego, CA, USA) platform using paired-end 2 × 300 bp reads. DNA extraction was carried out using the MagSi-DNA Stool kit (Magtivio, Nijverheidsweg, The Netherlands). Raw reads underwent quality filtering including trimming at a quality threshold corresponding to a Phred score of Q23 (error probability 0.005), removal of reads containing ambiguous bases, and exclusion of reads shorter than 270 bp. Paired-end reads were merged using a minimum score of 60, with mismatch cost = 4, gap cost = 5, and no unaligned end mismatches permitted. Bioinformatics processing was performed using the CLC Genomics Workbench (Qiagen, Hilden, Germany). Operational taxonomic units (OTUs) were generated using a clustering threshold of 98.5% sequence similarity. Taxonomic assignment was conducted using the NCBI RefSeq database (laboratory-optimized reference dataset), with a minimum similarity threshold of 85% for annotation. Chimera detection was performed using a k-mer-based approach followed by alignment-based validation, and chimeric sequences were excluded from downstream analysis. A minimum sequencing depth of 16,000 merged reads per sample was required for inclusion.

The taxa included in the analyses reflect the complete panel of microbial targets reported by the external certified laboratory. No taxa were selectively excluded or added by the research team, and all reported taxa were included in the statistical analyses. Gut microbiota-derived bioindicators, including fecal pH, the Firmicutes/Bacteroidetes ratio, and the percentage of LPS-positive bacteria, were reported directly by the external certified laboratory as part of their standardized microbiota profiling assay. The percentage of LPS-positive bacteria reflects the cumulative relative abundance of Gram-negative bacterial taxa identified within the laboratory’s panel, given that LPS is a structural component of the outer membrane of Gram-negative bacteria. The Firmicutes/Bacteroidetes ratio was derived from the phylum-level relative abundance values as reported in the laboratory outputs.

Fecal short-chain fatty acids (lactate, acetate, propionate, and butyrate) were quantified in the same certified external laboratory that performed the gut microbiota analysis, using headspace gas chromatography–mass spectrometry (HS-GC-MS; PerkinElmer Clarus 680 gas chromatograph coupled with a Clarus SQ8 mass spectrometer and TurboMatrix headspace system; PerkinElmer, Waltham, MA, USA). Prior to analysis, stool samples were homogenized in a high-salt solution and acidified, followed by the addition of an internal standard (2-ethylbutyric acid). Samples were then subjected to headspace analysis under controlled temperature conditions, and chromatographic separation was achieved using a fatty acid–specific capillary column. Quantification was performed by normalizing analyte signal intensities to the internal standard, and short-chain fatty acids concentrations were expressed as µmol per gram of feces.

Anthropometric data were collected during routine clinical consultations at the nutrition and dietetics clinic from which the retrospective patient data were derived. At the time of the clinical visit, data on bowel habits and sleep duration were obtained using a standardized questionnaire. Stool frequency and consistency were recorded based on patient self-report, with stool form classified according to the Bristol Stool Form Scale. The study investigators were not involved in sample processing, and all data were retrospectively extracted from the patients’ medical records, reflecting real-world clinical practice.

### 2.3. Statistical Analysis

Bacterial relative abundance data, expressed as percentages of total microbial community composition were received from the external certified laboratory and were used directly for taxon-level statistical comparisons, without additional transformation or normalization. Data are described as means ± standard deviation, median and quartiles (min-max) or in number (n) and percentage (%). Differences between study groups were determined using Kruskal–Wallis-test for non-parametric data and student *t*-test for parametric data. For categorical variables differences between study groups were analyzed by chi squared test. For pairwise comparison between groups Bonferroni method was used for multiple tests correction. Statistical analyses were performed using SPSS version 31.0 (IBM SPSS Statistics, Ehningen, Germany). All tests for significance were two sided, and a *p*-value of <0.05 was considered statistically significant. Kruskal–Wallis testing on relative abundances with Bonferroni correction was retained as the primary method for taxon-level comparisons given the nature of the input data, the absence of raw sequencing count data required by count-based compositional methods, and the limited sample size of the study, which reduces the statistical power available for more complex analytical frameworks. As an alternative differential abundance method, taxon-level analysis was additionally performed using LinDA (Linear Models for Differential Abundance Analysis), implemented via the MicrobiomeStat R package (version 1.18) in the R statistical environment (version 4.5.3) [22]. LinDA applies centered log-ratio transformation to relative abundance data with bias correction for compositional effects and accepts proportion data as input, making it suitable for the data format available in this study. Analyses were performed at the phylum and genus levels. To obtain all pairwise comparisons between study groups, the LinDA model was run iteratively with each group specified as the reference category. A minimum prevalence filter of 10% was applied prior to testing. Multiple testing correction was performed using the Benjamini–Hochberg false discovery rate (FDR) procedure, with a significance threshold of FDR-adjusted *p* < 0.05.

For additional exploratory analyses of gut microbial community structure, the relative abundance data received from the external laboratory were further processed and analyzed using the MicrobiomeAnalyst web platform (version 2.0) [23]. Prior to analysis, an initial filtering step was applied to remove features present in less than 10% of the samples. Features exhibiting low variability (lowest 10% based on inter-quantile range) across samples were also excluded. As the input data consisted of relative abundance values, no additional normalization was applied prior to diversity assessment. Alpha diversity was assessed using the Shannon index and the Simpson index, calculated at genus level using the MicrobiomeAnalyst platform (version 2.0), applied to the same dataset used for beta diversity analyses. Differences between study groups were evaluated using the Kruskal–Wallis test, with post hoc pairwise comparisons performed using the Dunn test with Benjamini–Hochberg correction. Beta diversity assessment was performed using Principal Coordinates Analysis (PCoA) constructed on the basis of Bray–Curtis dissimilarity to visualize differences in microbial community composition between study groups. Statistical significance of between-group differences was evaluated using permutational multivariate analysis of variance (PERMANOVA) applied to Bray–Curtis, Jaccard, and Jensen–Shannon distance measures (*p* < 0.05). In addition to the global PERMANOVA test, pairwise comparisons between all group combinations were performed. Multiple testing correction for pairwise contrasts was applied using the Benjamini–Hochberg false discovery rate (FDR) procedure. The normalization procedures described for the MicrobiomeAnalyst-based analyses were applied exclusively for beta and alpha diversity assessments and were not used for taxon-level comparisons. In addition, gut microbiota–derived bioindicators reflecting compositional and functional characteristics of the intestinal microbiota (Firmicutes/Bacteroidetes ratio, feces pH, LPS-positive bacteria, butyrate production, lactate production, acetate/propionate production) were extracted from the microbiome analysis reports and analyzed as provided.

Spearman rank correlation analysis was performed to assess associations between microbial taxa relative abundance and clinical and functional parameters, including short-chain fatty acid concentrations, serum lipid profile, BMI, and vitamin D levels. Multiple testing correction was applied using the Benjamini–Hochberg false discovery rate (FDR) procedure, with a significance threshold of FDR-adjusted *p* < 0.05. All correlation analyses were performed in the R statistical environment (version 4.5.3) using the cor.test function with the spearman method [24].

## 3. Results

### 3.1. Characteristics

Table 1 summarizes the baseline characteristics of the study population by group. Of the 59 participants included in the analysis, 8 were assigned to the HC group, 13 to the AG, 11 to the AI, and 27 to the IBS group. In the AI (*n* = 11), the most frequent condition was Hashimoto’s thyroiditis (*n* = 5), followed by psoriasis vulgaris (*n* = 2), combined autoimmune thyroiditis and psoriasis vulgaris (*n* = 2), rheumatoid arthritis (*n* = 1), and autoimmune thyroiditis associated with autoimmune gastritis (*n* = 1). While the HC and AI groups exhibited body mass index (BMI) values within the normal range, the AG presented BMI values indicative of underweight, whereas the irritable bowel syndrome group showed BMI values in the overweight range. These differences in BMI across groups were statistically significant (*p* < 0.001). Moreover, pairwise comparisons between study groups revealed statistically significant differences in terms of BMI between the AG and the AI (adjusted *p* = 0.001), as well as between the AG and the IBS [18.1 (16.7; 19.4) in AG vs. 24.3 (22.8; 28.1) in the AI; adjusted *p* < 0.001].

No statistically significant differences were observed between study groups in terms of age, sex, number of hours of sleep per night, fasting glycemia, high-density lipoprotein cholesterol (HDL), low-density lipoprotein cholesterol (LDL), serum triglyceride levels, or weekly bowel movement. However, a significant difference was identified for total cholesterol across groups (Kruskal–Wallis test, *p* = 0.039). Post hoc pairwise analysis revealed that the healthy control group exhibited significantly lower total cholesterol levels compared to the autoimmune diseases group (adjusted *p* = 0.044). Moreover, serum vitamin D levels differed significantly between study groups (Kruskal–Wallis test, *p* = 0.005). Post hoc pairwise comparisons revealed that the AI group exhibited significantly lower serum vitamin D levels compared to the HC group [20.8 (14.1; 29.7) vs. 40.8 (35.7; 41.2), adjusted *p* = 0.005]. No other pairwise comparisons reached statistical significance after adjustment for multiple testing.

### 3.2. Microbiome Analysis

#### 3.2.1. Phylum Level

**Figure 1:**
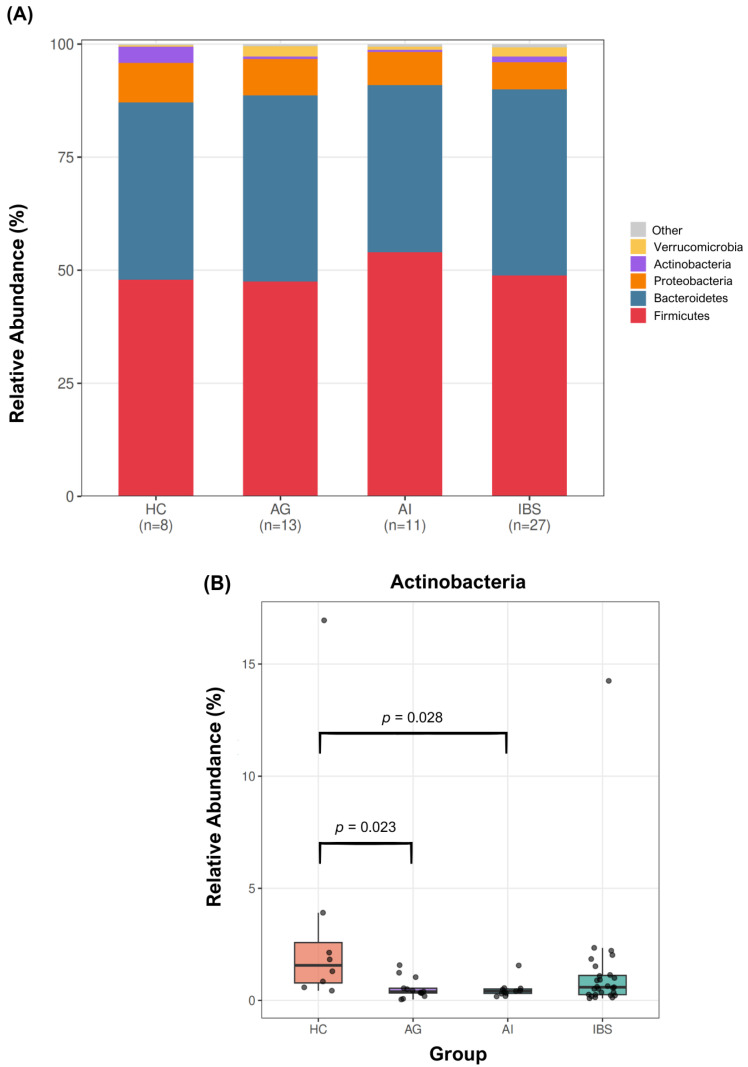
Phylum-level microbial relative abundance across study groups. (**A**) Stacked bar plot showing the relative abundance (%) of the major microbial phyla across study groups (HC, AG, AI, and IBS); sample sizes are indicated below each group, and low-abundance taxa are grouped as ‘Other’; (**B**) Boxplot of Actinobacteria relative abundance with pairwise significance brackets (HC vs. AG: adjusted *p* = 0.023; HC vs. AI: adjusted *p* = 0.028). Individual data points are shown. HC, healthy controls; AG, anxiety group; AI, autoimmune group; IBS, irritable bowel syndrome group. Statistical significance was assessed using the Kruskal–Wallis test with Bonferroni correction for pairwise comparisons. LinDA analysis confirmed the significant reduction in Actinobacteria in the AG compared to HC (log2FoldChange = −1.897, FDR-adjusted *p* = 0.049).

Analysis of the gut microbiota revealed differences in microbial composition across the study groups. As presented in Figure 1, at the phylum level, Actinobacteria differed significantly among groups (Kruskal–Wallis test, *p* = 0.015), with higher relative abundances observed in HC [1.5 (0.6–3.5)] and the lowest levels in the AG [0.3 (0.2–0.8)] (Table 2). Post hoc pairwise comparisons revealed significantly higher relative abundances of Actinobacteria in HC compared to both the AI (adjusted *p* = 0.028) and the AG (adjusted *p* = 0.023). No significant differences were observed among study groups for the remaining bacterial phyla analyzed. Moreover, LinDA analysis confirmed the significant reduction in Actinobacteria in the AG compared to HC (log2FoldChange = −1.897, FDR-adjusted *p* = 0.049), representing the only taxon to reach FDR-corrected significance across all LinDA pairwise comparisons (Supplementary Table S1).

#### 3.2.2. Genus Level

**Figure 2:**
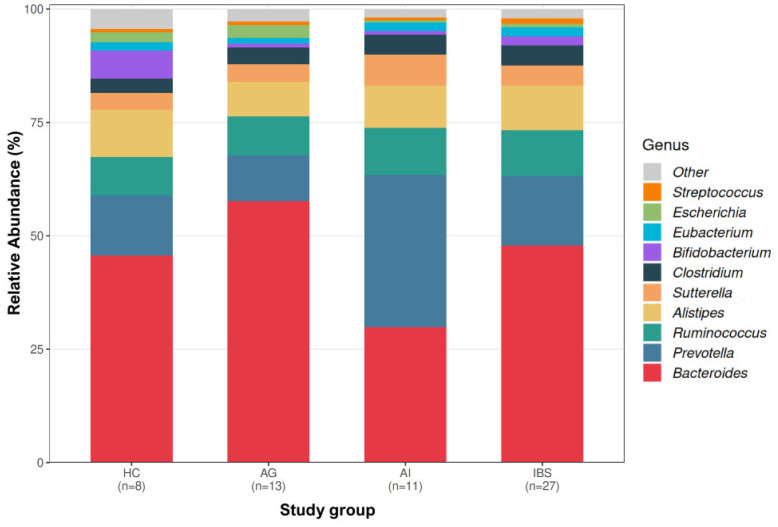
Genus-level microbial composition across study groups. Stacked bar plot showing the mean relative abundance of the top 10 genera per group, normalized to 100% of genus-level abundance. Remaining genera are grouped as ‘Other’. HC, healthy controls; AG, anxiety group; AI, autoimmune group; IBS, irritable bowel syndrome group.

**Figure 3:**
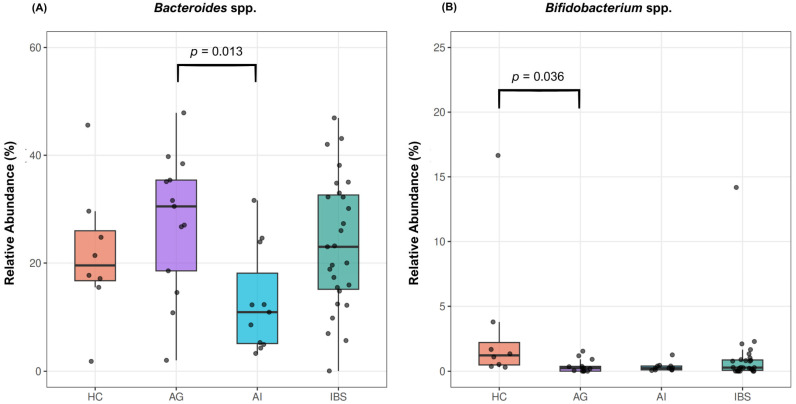
Relative abundance of key genera across study groups. (**A**) *Bacteroides* spp.—significantly higher in the AG compared to the AI group (adjusted *p* = 0.013). (**B**) *Bifidobacterium* spp.—significantly higher in the HC group compared to the AG (adjusted *p* = 0.036). Boxes represent median and interquartile range; whiskers extend to 1.5 × IQR; individual data points are presented. HC, healthy controls; AG, anxiety group; AI, autoimmune group; IBS, irritable bowel syndrome group. Statistical significance was assessed using the Kruskal–Wallis test with Bonferroni correction for pairwise comparisons.

The overall genus-level microbial composition across study groups is illustrated in Figure 2, showing the relative abundance of the top 10 genera per group. Comparative analysis (Kruskal–Wallis test with Bonferroni correction) identified significant differences involving two bacterial genera across the study groups (Table 3), namely *Bacteroides* spp. (*p* = 0.023) and *Bifidobacterium* spp. (*p* = 0.038) (Figure 3). The HC group exhibited the highest relative abundance of *Bifidobacterium* spp. compared to the other groups [1.22 (0.49; 2.21)]. Post hoc pairwise comparisons indicated significantly higher relative abundances of *Bifidobacterium* spp. in the HC group compared to the AG (adjusted *p* = 0.036). In contrast, the highest relative abundance of *Bacteroides* spp. was observed in the AG, whereas the lowest levels were detected in the AI [60.19 (53.54–73.41) vs. 26.64 (13.63–52.43)]. These findings were supported by post hoc pairwise analysis, which indicated significantly higher relative abundances of *Bacteroides* spp. in the AG compared to the AI (adjusted *p* = 0.013). No other pairwise comparisons remained statistically significant after adjustment for multiple testing. In the LinDA analysis, no genus-level taxon reached statistical significance after FDR correction. However, *Bifidobacterium* spp. showed consistently lower abundance in AG and AI compared to HC at nominal significance, supporting the directionality of the Kruskal–Wallis findings (Supplementary Table S1).

#### 3.2.3. Alpha Diversity, Beta Diversity and Other Bioindicators

**Figure 4:**
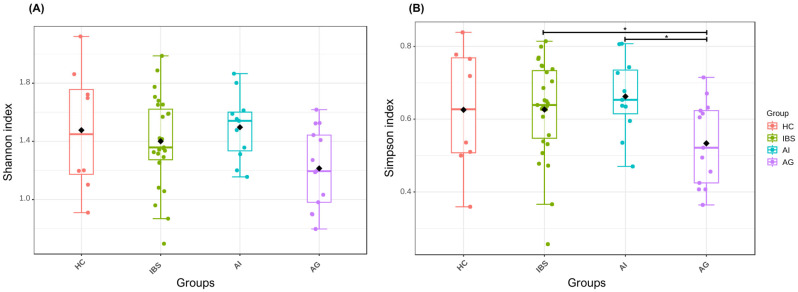
Alpha diversity indices across study groups. (**A**) Shannon index (Kruskal–Wallis H = 5.764, *p* = 0.124). (**B**) Simpson index (Kruskal–Wallis H = 7.731, *p* = 0.052); asterisks indicate FDR-adjusted significant pairwise differences *p* < 0.05 (IBS vs. AG: FDR = 0.042; AI vs. AG: FDR = 0.042). Diamonds represent group means. HC, healthy controls; AG, anxiety group; AI, autoimmune group; IBS, irritable bowel syndrome group.

As shown in Table 4, alpha diversity assessed by the Shannon index did not differ significantly between study groups (Kruskal–Wallis H = 5.764, *p* = 0.124). Post hoc pairwise comparisons did not identify any significant differences after FDR correction. Similarly, the global Kruskal–Wallis test for the Simpson index did not reach statistical significance (H = 7.731, *p* = 0.052). However, post hoc pairwise comparisons revealed significantly lower Simpson index values in the AG compared to both the IBS group (*p* = 0.014, FDR = 0.042) and the AI group (*p* = 0.009, FDR = 0.042), suggesting reduced evenness in microbial community structure in the anxiety group. Alpha diversity results are presented in Figure 4. Significant differences between groups were observed for lactate production (*p* = 0.015) and acetate/propionate ratio (*p* = 0.039). Post hoc pairwise comparisons revealed that lactate production was significantly higher in the HC group compared to the AI (*p* = 0.014) and AG (*p* = 0.038). For the acetate/propionate ratio, despite a significant overall group effect identified by the Kruskal–Wallis test, post hoc pairwise analyses did not retain statistical significance after correction for multiple comparisons. No significant differences were detected for Firmicutes/Bacteroidetes ratio, butyrate production, or LPS-positive bacteria.

**Figure 5:**
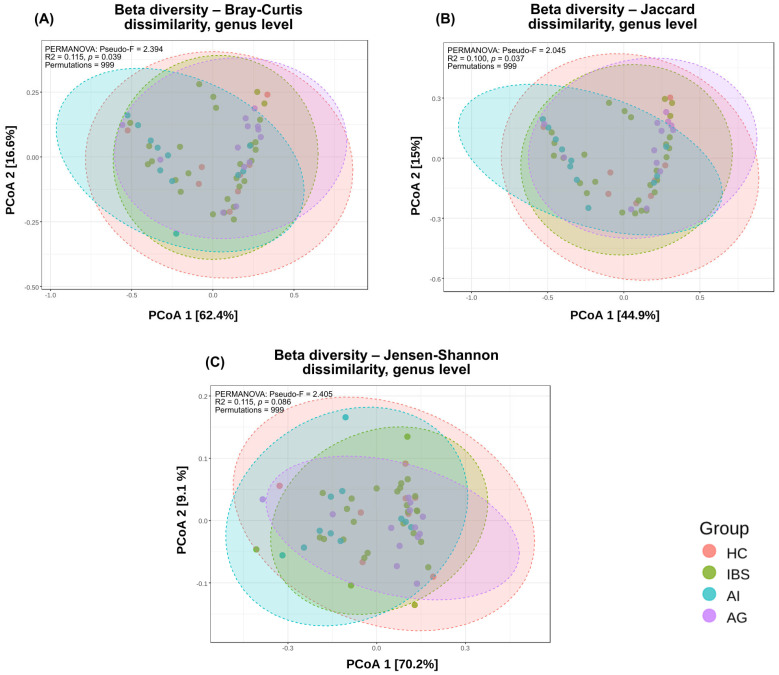
Beta diversity assessed by principal coordinates analysis (PCoA) at genus level. (**A**) Bray–Curtis dissimilarity (PERMANOVA: Pseudo-F = 2.395, R^2^ = 0.116, *p* = 0.039); (**B**) Jaccard dissimilarity (PERMANOVA: Pseudo-F = 2.045, R^2^ = 0.100, *p* = 0.037); (**C**) Jensen–Shannon dissimilarity (PERMANOVA: Pseudo-F = 2.406, R^2^ = 0.116, *p* = 0.086). Percentage of variance explained by each principal coordinate for Jensen–Shannon dissimilarity was recalculated using the cmdscale function in R (vegan package), as the platform used for visualization produced inconsistent eigenvalue estimates for this distance metric. All pairwise comparisons were performed with Benjamini–Hochberg FDR correction; the AI vs. AG comparison reached statistical significance after correction for Bray–Curtis dissimilarity (Pseudo-F = 6.327, R^2^ = 0.223, FDR-adjusted *p* = 0.048). All analyses based on 999 permutations.

As illustrated in Figure 5, beta diversity assessed by PCoA based on Bray–Curtis dissimilarity at genus level revealed overlapping distributions across the four study groups, with no clear visual separation. PERMANOVA analysis indicated statistically significant differences in overall microbial community composition between groups for two of three distance metrics: Bray–Curtis (Pseudo-F = 2.395, R^2^ = 0.116, *p* = 0.039) and Jaccard (Pseudo-F = 2.045, R^2^ = 0.100, *p* = 0.037), while Jensen–Shannon dissimilarity did not reach statistical significance (Pseudo-F = 2.406, R^2^ = 0.116, *p* = 0.086). Pairwise PERMANOVA comparisons based on Bray–Curtis dissimilarity identified a significant difference between the AI and AG groups (Pseudo-F = 6.327, R^2^ = 0.223, FDR-adjusted *p* = 0.048), while no other pairwise comparisons reached statistical significance after FDR correction.

**Figure 6:**
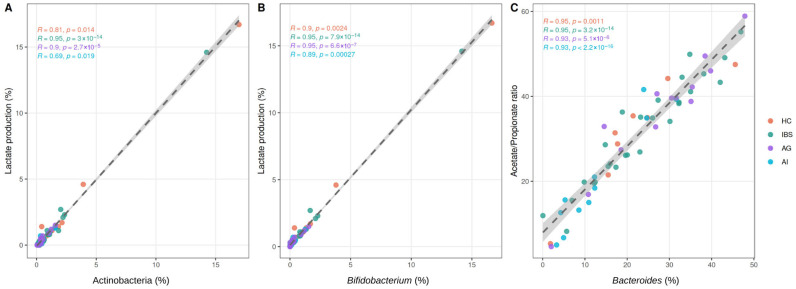
Spearman rank correlations between microbial taxa and functional parameters. (**A**) Actinobacteria relative abundance and lactate production (r = 0.910, FDR-adjusted *p* < 0.001); (**B**) *Bifidobacterium* spp. relative abundance and lactate production (r = 0.941, FDR-adjusted *p* < 0.001); (**C**) *Bacteroides* spp. relative abundance and acetate/propionate ratio (r = 0.955, FDR-adjusted *p* < 0.001). Each point represents one participant, colored by study group. The dashed line represents the overall linear regression fit. Spearman correlation coefficients and *p*-values per group are shown in the upper left corner of each panel.

Spearman correlation analysis revealed significant associations between microbial taxa and functional parameters across the cohort. The strongest correlations were observed between *Bifidobacterium* spp. and lactate production (r = 0.941, FDR-adjusted *p* < 0.001), *Bacteroides* spp. and the acetate/propionate ratio (r = 0.955, FDR-adjusted *p* < 0.001), and Actinobacteria and lactate production (r = 0.910, FDR-adjusted *p* < 0.001). Significant negative associations were identified between Actinobacteria and *Bifidobacterium* spp. abundance and total cholesterol levels (r = −0.383 and r = −0.409, respectively; FDR-adjusted *p* < 0.05). Full Spearman correlation results are presented in Supplementary Table S2, and the correlation matrix is illustrated in Supplementary Figure S1. Scatter plots for the three strongest associations are shown in Figure 6.

## 4. Discussion

The present study provides a comparative overview of gut microbiota patterns across individuals with irritable bowel syndrome, anxiety disorders, and autoimmune diseases in a Romanian population. The main findings indicate that, although global microbial diversity did not differ significantly between groups, distinct taxonomic and functional features were observed. HC group was characterized by higher relative abundances of Actinobacteria and *Bifidobacterium* spp. and by increased lactate production, whereas the AG showed the highest abundance of *Bacteroides* spp. In addition, the AI group exhibited significantly lower serum vitamin D levels and higher total cholesterol compared with HC. Together, these results may indicate that disease-specific microbial signatures could be present even in the absence of major shifts in overall community structure.

Although global alpha diversity did not differ significantly between groups when assessed by the Shannon index, the Simpson index revealed significantly lower microbial evenness in the AG compared to both the IBS and AI groups, suggesting a less balanced community structure in anxiety-related conditions. Regarding beta diversity, PERMANOVA analysis identified statistically significant differences in overall microbial community composition between groups, with pairwise analysis further highlighting a significant separation between the AI and AG groups. These findings may indicate that, although broad diversity metrics may not capture all relevant differences, targeted taxonomic and functional analyses can highlight potential microbial patterns. However, these observations remain exploratory and should be interpreted with caution, consistent with previous human microbiome studies reporting compositional and functional variability in the absence of major changes in global diversity indices. For example, Butler et al. showed that, although alpha diversity did not differ between patients with social anxiety disorder and HC, significant compositional and functional alterations were observed, with increased abundances of the genera *Anaeromassillibacillus* and *Gordonibacter* in patients and *Parasutterella* predominating in controls [25]. Similarly, in another study, rheumatoid arthritis patients showed no differences in alpha diversity compared with controls, but exhibited significant beta-diversity, taxonomic and metabolomic alterations, including depletion of Bacteroidetes and *Faecalibacterium* and enrichment of Verrucomicrobia, Proteobacteria, *Lactobacillus*, *Streptococcus* and *Akkermansia* [26].

In contrast to our findings, a large meta-analysis of 1167 fecal 16S rRNA samples showed significantly lower alpha diversity in patients with IBS compared with HC [2]. The authors also identified 26 bacterial genera and 12 predicted functional pathways that differentiated the two groups and reported that lower abundances of Ruminococcaceae and Christensenellaceae families were associated with reduced vitamin B6 and tyrosine metabolism in IBS patients [2]. Several cohort studies have also reported compositional alterations in IBS, most commonly a reduced abundance of butyrate-producing bacteria such as *Faecalibacterium* and *Roseburia*, together with an increase in Proteobacteria. Several cohort studies have reported compositional alterations in IBS, most commonly marked by a reduced abundance of butyrate-producing bacteria, while microbiota profiles vary between IBS subtypes, suggesting that these microbial changes are not uniform across patients [27,28]. In line with these observations, a study including 113 IBS patients and 66 healthy controls showed that lower microbial diversity was associated with a reduced abundance of butyrate-producing taxa, particularly members of the Ruminococcaceae, unclassified Clostridiales and Erysipelotrichaceae families, with this reduction being more pronounced in diarrhoea-predominant IBS (IBS-D) and alternating-type IBS (IBS-M) patients. Moreover, untreated IBS patients also had fewer methanogenic microorganisms, as Methanobacteria (phylum Euryarchaeota) were enriched in controls, and several taxa were correlated with the severity of flatulence and abdominal pain [27]. Further evidence for the heterogeneity of IBS comes from a study that identified distinct microbiota-based subgroups within the patient cohort of IBS patients. IBS-D patients showed a microbiota composition more similar to that of healthy controls, whereas constipation-predominant IBS (IBS-C) and IBS-M were characterized by more pronounced dysbiosis, with an increased abundance of Firmicutes-associated taxa and a depletion of Bacteroidetes-related taxa [28]. Moreover, specific microbial signatures were associated with colonic transit time as well as with the severity of depressive symptoms, suggesting that microbiota composition reflects both physiological and clinical differences between IBS subtypes [28]. A systematic review further supports the presence of compositional alterations in IBS, reporting increased abundances of Enterobacteriaceae, Lactobacillaceae and *Bacteroides*, together with reduced *Faecalibacterium*, *Faecalibacterium prausnitzii*, *Bifidobacterium* and uncultured Clostridiales. Importantly, microbial diversity was reported as either decreased or unchanged across the included studies, highlighting the inconsistency of diversity findings in IBS [29]. Overall, the available evidence indicates that IBS is characterized by reproducible taxonomic and functional alterations, despite inconsistent findings regarding microbial diversity. This heterogeneity, together with the influence of IBS subtypes, may partly explain the lack of diversity differences in our cohort and supports the need for a more targeted analysis of specific microbial signatures.

Although *Bacteroides* spp. showed significantly higher abundance in AG compared to AI by Kruskal–Wallis analysis, this finding was not confirmed in the LinDA analysis and should be interpreted with caution as an exploratory observation. Higher relative abundance of *Bacteroides* spp. in the AG compared to the AI may reflect shifts in microbial ecology relevant to the gut–brain axis. *Bacteroides*-dominated community configurations (often described as “Bacteroides enterotype”) have been associated with long-term dietary patterns characterized by higher intake of animal protein and fat, a pattern frequently discussed in the context of Westernized diets [30,31]. In parallel, *Bacteroides*-rich ecosystems are often associated with distinct microbial metabolic capacities, including processes that influence bile acid metabolism, molecules that are involved not only in lipid digestion but also act as signaling mediators with important immunometabolic effects [32,33]. The interplay between bile acid metabolism and the gut microbiota represents an important component of the gut–brain axis. Members of the genus *Bacteroides*, which are among the dominant taxa in the human intestine, contribute to the biotransformation of primary bile acids synthesized in the liver into secondary bile acids [34]. These microbially derived metabolites can modulate host physiology through several mechanisms, including the regulation of neuroinflammatory pathways, maintenance of blood–brain barrier integrity, and the modulation of neurotransmitter signaling [34]. Beyond bile acid metabolism, a growing body of evidence indicates that the gut microbiota can modulate the availability of neuroactive compounds and their precursors, including metabolites derived from tryptophan as well as other intermediates involved in neurotransmitter synthesis. These microbiota–host metabolic interactions represent biologically plausible pathways through which shifts in microbial composition may be linked to mechanisms relevant to anxiety [35,36]. Moreover, the lower relative abundance of *Bifidobacterium* spp. observed in the AG compared with HC group is in line with human psychobiotic studies reporting that specific *Bifidobacterium longum* strains can attenuate stress-related responses, including cortisol levels and perceived stress [37,38]. Although causal relationships cannot be inferred from our data, this pattern may reflect a lower abundance of bifidobacteria, which could weaken their protective involvement in stress resilience, neuroendocrine regulation, and intestinal barrier function in individuals with anxiety.

In our AI, the lower abundance of the phylum Actinobacteria together with reduced serum vitamin D levels may reflect a less favorable immunoregulatory environment [39]. Moreover, no significant reduction in Actinobacteria was observed in the IBS group, which may reflect the well-documented heterogeneity of IBS-associated microbiota patterns across subtypes and cohorts, as well as the lack of IBS subtype stratification in the present study [4,6,30]. Members of the Actinobacteria phylum, particularly *Bifidobacterium* spp., have been shown to promote regulatory T-cell differentiation, enhance epithelial barrier function and reduce pro-inflammatory cytokine production [39]. A decreased abundance of the genus *Bifidobacterium* spp. has been reported in several immune-mediated disorders, including inflammatory bowel disease [40], rheumatoid arthritis [26] and psoriasis [16], suggesting a potential link with impaired immune tolerance. In parallel, the hypovitaminosis D observed in our AI cohort is consistent with the well-established role of vitamin D in immune homeostasis, as this hormone modulates dendritic cell maturation, promotes regulatory T-cell responses and suppresses Th1- and Th17-mediated inflammation [41]. Thus, the concomitant reduction in the abundance of the phylum Actinobacteria and in serum vitamin D levels may indicate a microbiota profile with a lower capacity to maintain immune tolerance and to counteract pro-inflammatory signaling. Moreover, the significantly lower serum vitamin D levels observed in the AI group, despite the relatively young age of participants, may reflect impaired vitamin D metabolism secondary to chronic immune activation rather than age-related decline in cutaneous synthesis [41]. Several autoimmune conditions represented in this cohort, including Hashimoto’s thyroiditis, have been specifically associated with hypovitaminosis D, potentially mediated by altered vitamin D receptor signaling and increased metabolic consumption during immune-mediated inflammatory processes [14,41].

In our cohort, lactate levels were highest in HC and reduced in both the AG and AI, whereas the other metabolites did not differ significantly between groups. The higher lactate production observed in HC group supports the concept of a metabolically more active and functionally interconnected microbial ecosystem. Lactate represents a central intermediate in microbial cross-feeding, being converted by specific anaerobic bacteria into butyrate, a short-chain fatty acid with well-established roles in maintaining epithelial integrity, promoting regulatory immune responses and providing an energy source for colonocytes [42,43]. In this context, increased lactate availability may reflect a more functionally integrated microbial trophic network, supporting enhanced substrate exchange between lactate-producing and butyrate-producing bacteria [44]. Conversely, the lower lactate levels observed in the AG and AI group may indicate impaired metabolic interactions between microbial taxa that normally cooperate in short-chain fatty acid production [45], reflecting a less functionally integrated gut ecosystem rather than a simple decrease in diversity. These observations were further supported by Spearman correlation analysis in our analysis, which confirmed strong positive associations between Actinobacteria abundance and lactate production and between *Bifidobacterium* spp. abundance and lactate production across the entire cohort, suggesting a mechanistic link between the reduced abundance of these taxa in the AG and AI groups and the impaired lactate production capacity observed in these conditions. These alterations may have downstream effects on epithelial homeostasis and immune regulation [46,47]. Moreover, the absence of significant differences in the Firmicutes/Bacteroidetes ratio despite detectable functional metabolic alterations supports the concept that metabolic dysbiosis can occur independently of broad phylum-level compositional shifts, as lactate production and the acetate/propionate ratio are driven by specific cross-feeding networks rather than by global changes in dominant phyla [44,45].

Taken together, these findings highlight the presence of disease-related microbial and functional patterns despite the absence of major shifts in global diversity, supporting the relevance of integrative microbiome research in complex chronic disorders.

## 5. Strengths and Limitations

This study has several strengths, including its integrative design and the simultaneous evaluation of functional gastrointestinal, anxiety-related, and autoimmune disorders in a cohort from Romania. However, some limitations should be acknowledged. First, the cross-sectional and observational design precludes any inference of causality and may be subject to residual confounding and selection bias compared with prospective longitudinal approaches. A further limitation concerns the nature of the microbiota data available to the research team. As gut microbiota analyses were performed by a certified external laboratory using a proprietary sequencing and bioinformatics pipeline, the authors had access exclusively to processed relative abundance outputs as provided in the certified laboratory reports. Raw amplicon sequencing files were not transferred to the research team and could therefore not be deposited in a public repository. A further limitation concerns the interpretation of taxon-level differential abundance findings, particularly the discrepancy between the Kruskal–Wallis analysis and the LinDA alternative differential abundance method. Although both Kruskal–Wallis testing and LinDA were applied, several taxa that appeared significant in the Kruskal–Wallis analysis did not remain significant after multiple-testing adjustment in LinDA, a discrepancy likely attributable to the limited sample size and unequal distribution of participants across groups, particularly the low number of healthy controls, which reduced the statistical power available for robust differential abundance inference. Notably, only the reduction in Actinobacteria in the AG compared to HC reached FDR-corrected significance in the LinDA analysis, representing the sole finding confirmed by both statistical approaches. Accordingly, all taxon-level results should be regarded as exploratory and hypothesis-generating rather than definitive. This imbalance in group sizes is partly explained by the clinical recruitment setting, where individuals undergoing microbiota testing were more frequently patients with existing medical conditions than healthy subjects. Another limitation is the heterogeneity within the disease groups. In the IBS group, analyses were not stratified according to clinical subtypes, which may present distinct microbial and metabolic profiles [28]. Furthermore, stool consistency was assessed by patient self-report at a single time point, which may not capture the temporal variability characteristic of IBS, and the absence of IBS subtype stratification may have attenuated detectable differences in bowel habits across groups [27]. Similarly, in the AI, thyroid function status (euthyroid versus hypothyroid, for example) was not considered, although it may influence both metabolic parameters and gut microbiota composition [14]. In addition, although major clinical and biochemical parameters were evaluated, other potential confounding factors, such as detailed dietary intake and stress-related variables, were not comprehensively controlled for and may have contributed to interindividual variability in microbial composition. Although BMI differed significantly between study groups, the primary taxon-level analyses were not adjusted for this covariate due to the constraints of the non-parametric statistical framework employed, and residual confounding by BMI cannot be fully excluded. Finally, as microbiota profiles were derived from a standardized commercial 16S rRNA sequencing panel reflecting the diagnostic options available on the Romanian market, the reported taxa are limited to those included in the laboratory’s predefined panel. Consequently, microbial features outside this panel, including potentially relevant taxa or strain-level variants, were not captured, which may have limited the detection of condition-specific microbial signatures. Furthermore, the data provide information primarily on microbial composition rather than direct measurement of microbial metabolic activity, and do not allow discrimination at the strain level, which may mask functionally relevant differences between taxa.

## 6. Conclusions

Overall, the present study provides exploratory and hypothesis-generating evidence of potential disease-associated microbial and functional patterns across individuals with IBS, anxiety disorders, and autoimmune diseases in a Romanian cohort. Among the main findings, reduced relative abundance of Actinobacteria in the anxiety group compared to healthy controls was the most robust observation, being confirmed by both Kruskal–Wallis analysis and LinDA analysis. Other taxonomic and functional differences identified at the genus level and in short-chain fatty acid profiles should be interpreted with caution, given the limited sample size, the lack of access to raw sequencing data, and the constraints associated with the use of processed microbiome outputs. Accordingly, these results may reflect preliminary patterns rather than definitive microbial signatures. Taken together, these findings may contribute to the generation of hypotheses regarding gut–brain–immune interactions and provide population-specific data from an underrepresented Eastern European cohort [19,20]. Future research should prioritize longitudinal and mechanistic approaches with access to raw sequencing data, larger sample sizes, and compositionally aware statistical frameworks to validate and extend these preliminary observations.

## Figures and Tables

**Table 1:** Patients characteristics and serum parameters by study groups.

Characteristics	HC *N* = 8	AG Anxiety *N* = 13	AI Autoimmune *N* = 11	IBS *N* = 27	*p*-Value
Demographic and lifestyle parameters					
Age, years	32.9 ± 11.2	39.5 ± 11.2	44.7 ± 9.6	40.2 ± 10.7	0.138
Women, n (%)	4 (50.0%)	9 (69.2%)	8 (72.7%)	19 (70.4%)	0.711
BMI, kg/m^2^	20.9 (19.9; 23,1)	18.1 (16.7; 19.4)	24.3 (22.8; 28.1)	25.4 (21.8; 30.4)	<0.001
Sleep, h/night	7.7 (7.5; 8.0)	7.0 (5.0; 8.0)	6.5 (5.5; 7.0)	7.0 (5.0; 8.0)	0.166
Serum and stool parameters					
Glycemia, mg/dL	86.3 ± 9.4	90.7 ± 9.7	89.9 ± 13.4	91.2 ± 10.8	0.713
Total cholesterol, mg/dL	183.5 (176.0; 184.8)	182.0 (158.5; 227.7)	215.0 (188.0; 255.0)	197.0 (189.0; 221.0)	0.039
HDL cholesterol, mg/dL	52.9 (45.7; 73.2)	63.0 (49.5; 79.3)	56.0 (45.5; 66.0)	63.0 (45.0; 76.9)	0.643
LDL cholesterol, mg/dL	126.5 (102.0; 137.9)	113.0 (102.0; 137.6)	169.0 (98.0; 210.0)	124.0 (111.0; 155.0)	0.224
Tryglicerides, mg/dL	73.5 (47.8; 96.5)	70.0 (39.0; 77.5)	129.0 (74.0; 160.0)	95.0 (59.0; 149.)	0.083
Vitamin D, ng/mL	40.8 (35.7; 41.2)	29.0 (22.0; 59.6)	20.8 (14.1; 29.7)	28.4 (19.4; 36.4)	0.005
Bristol stool scale	4.0 (3.0; 4.0)	4.0 (2.0; 6.0)	2.0 (2.0; 3.0)	2.0 (2.0; 4.0)	0.205
Weekly bowel movement frequency	7.0 (7.0; 11.5)	7.0 (4.5; 7.0)	6.0 (4.0; 7.0)	7.0 (5.0; 7.0)	0.091

HC = healthy control; AG = anxiety group; AI = autoimmunity group, IBS = irritable bowel syndrome group. HDL = high-density lipoprotein, LDL = low-density lipoprotein.

**Table 2:** Phylum level-relative abundance.

Phylum	HC *N* = 8	AG Anxiety *N* = 13	AI Autoimmune *N* = 11	IBS *N* = 27	*p*-Value
Firmicutes	47.71 (42.26; 52.59)	48.8 (41.27; 50.07)	49.65 (46.8; 57.32)	49.64 (39.89; 55.47)	0.533
Bacteroidetes	39.13 (33.85; 40.52)	38.29 (37.62; 43.24)	35.14 (33.93; 40.92)	39.86 (34.3; 47.01)	0.612
Proteobacteria	6.84 (5.07; 9.03)	7.0 (4.6; 10.12)	5.68 (4.99; 8.62)	5.62 (4.07; 6.77)	0.433
Actinobacteria	1.56 (0.78; 2.58)	0.39 (0.33; 0.54)	0.41 (0.32; 0.51)	0.59 (0.26; 1.11)	0.015
Verrucomicrobia	0.08 (0.0; 0.49)	0.49 (0.03; 3.42)	0.1 (0.01; 0.66)	0.34 (0.01; 1.55)	0.483
Fusobacteria	0.0 (0.0; 0.0)	0.0 (0.0; 0.0)	0.0 (0.0; 0.0)	0.0 (0.0; 0.0)	0.729
Cyanobacteria	0.07 (0.03; 0.13)	0.05 (0.01; 0.19)	0.05 (0.03; 0.08)	0.03 (0.01; 0.22)	0.835
Euryarchaeota	0.0 (0.0; 0.01)	0.0 (0.0; 0.0)	0.0 (0.0; 0.0)	0.0 (0.0; 0.0)	0.338
Tenericutes	0.01 (0.0; 0.02)	0.01 (0.01; 0.16)	0.03 (0.0; 0.7)	0.02 (0.0; 0.07)	0.542

HC = healthy control; AG = anxiety group; AI = autoimmunity group, IBS = irritable bowel syndrome group. Statistical significance was assessed using the Kruskal–Wallis test with Bonferroni correction for pairwise comparisons.

**Table 3:** Genus level-relative abundance.

Genus	HC *N* = 8	AG Anxiety *N* = 13	AI *N* = 11	IBS *N* = 27	*p*-Value
*Prevotella* spp.	0.02 (0.01; 8.34)	0.03 (0.0; 1.85)	17.07 (3.43; 22.7)	0.21 (0.01; 13.48)	0.165
*Desulfobacter* spp.	0.0 (0.0; 0.0)	0.0 (0.0; 0.0)	0.0 (0.0; 0.0)	0.0 (0.0; 0.0)	0.884
*Desulfovibrio* spp.	0.02 (0.0; 0.07)	0.02 (0.01; 0.06)	0.07 (0.02; 0.39)	0.04 (0.01; 0.12)	0.216
*Desulfuromonas* spp.	0.0 (0.0; 0.0)	0.0 (0.0; 0.0)	0.0 (0.0; 0.0)	0.0 (0.0; 0.0)	0.316
*Oscillibacter* spp.	0.06 (0.02; 0.1)	0.14 (0.01; 0.19)	0.09 (0.06; 0.33)	0.13 (0.05; 0.24)	0.481
*Alistipes* spp.	4.66 (3.01; 7.06)	3.29 (0.98; 5.59)	3.28 (1.12; 6.03)	4.56 (1.93; 5.85)	0.848
*Methanobrevibacter* spp.	0.0 (0.0; 0.01)	0.0 (0.0; 0.0)	0.0 (0.0; 0.0)	0.0 (0.0; 0.0)	0.386
*Methanobacteria*	0.0 (0.0; 0.01)	0.0 (0.0; 0.0)	0.0 (0.0; 0.0)	0.0 (0.0; 0.0)	0.353
*Citrobacter* spp.	0.0 (0.0; 0.16)	0.0 (0.0; 0.0)	0.0 (0.0; 0.0)	0.0 (0.0; 0.01)	0.130
*Enterobacter* spp.	0.0 (0.0; 0.33)	0.01 (0.0; 0.1)	0.0 (0.0; 0.02)	0.01 (0.0; 0.04)	0.654
*Escherichia* spp.	0.42 (0.03; 0.71)	0.1 (0.06; 0.91)	0.08 (0.04; 0.23)	0.1 (0.01; 0.48)	0.814
*Klebsiella* spp.	0.0 (0.0; 0.33)	0.0 (0.0; 0.12)	0.0 (0.0; 0.01)	0.01 (0.0; 0.03)	0.686
*Providencia* spp.	0.0 (0.0; 0.0)	0.0 (0.0; 0.0)	0.0 (0.0; 0.0)	0.0 (0.0; 0.0)	0.241
*Pseudomonas* spp.	0.0 (0.0; 0.0)	0.0 (0.0; 0.0)	0.0 (0.0; 0.0)	0.0 (0.0; 0.0)	0.671
*Serratia* spp.	0.0 (0.0; 0.0)	0.0 (0.0; 0.0)	0.0 (0.0; 0.0)	0.0 (0.0; 0.0)	0.519
*Sutterella* spp.	1.21 (0.41; 2.01)	1.72 (0.55; 4.03)	2.92 (1.24; 4.36)	1.6 (0.07; 3.2)	0.312
*Enterococcus* spp.	0.0 (0.0; 0.09)	0.0 (0.0; 0.01)	0.0 (0.0; 0.01)	0.0 (0.0; 0.01)	0.726
*Ruminococcus* spp.	3.81 (2.96; 5.08)	2.95 (2.54; 4.61)	3.34 (3.14; 4.96)	4.16 (3.0; 5.79)	0.511
*Eubacterium* spp.	0.73 (0.42; 0.84)	0.34 (0.04; 0.92)	0.64 (0.37; 0.8)	0.64 (0.2; 1.31)	0.531
*Bacteroides* spp.	19.56 (16.72; 25.99)	30.51 (18.55; 35.4)	10.92 (5.12; 18.12)	23.01 (15.15; 32.63)	0.023
*Dorea* spp.	0.3 (0.2; 0.42)	0.24 (0.09; 0.31)	0.14 (0.08; 0.27)	0.16 (0.13; 0.28)	0.346
*Bifidobacterium* spp.	1.22 (0.49; 2.21)	0.24 (0.01; 0.37)	0.24 (0.1; 0.4)	0.28 (0.08; 0.87)	0.038
*Lactobacillus* spp.	0.0 (0.0; 0.04)	0.0 (0.0; 0.01)	0.0 (0.0; 0.01)	0.01 (0.0; 0.02)	0.134
*Clostridium* spp.	1.2 (0.96; 1.44)	1.38 (0.65; 2.15)	1.27 (1.26; 1.94)	1.74 (0.94; 2.78)	0.623
*Streptococcus* spp.	0.31 (0.26; 0.41)	0.11 (0.06; 0.25)	0.2 (0.05; 0.3)	0.19 (0.09; 0.57)	0.378
*Candida* spp.	0.0 (0.0; 0.0)	0.0 (0.0; 0.0)	0.0 (0.0; 0.0)	0.0 (0.0; 0.0)	0.954

HC = healthy control; AG = anxiety group; AI = autoimmunity group, IBS = irritable bowel syndrome group. Statistical significance was assessed using the Kruskal–Wallis test with Bonferroni correction for pairwise comparisons.

**Table 4:** Alpha diversity, beta diversity and other bioindicators in the study groups.

Bioindicator	HC *N* = 8	AG Anxiety *N* = 13	AI *N* = 11	IBS *N* = 27	*p*-Value
Alpha diversity (Shannon index)	1.45 (1.17; 1.76)	1.19 (0.98; 1.44)	1.54 (1.33; 1.60)	1.36 (1.27; 1.62)	0.124
Alpha diversity (Simpson index)	0.63 (0.51; 0.77)	0.52 (0.42; 0.62)	0.65 (0.61; 0.73)	0.64 (0.55; 0.73)	0.052
Feces pH	7.00 (6.38; 7.00)	6.50 (6.50; 7.00)	6.50 (6.00; 6.50)	6.50 (6.50; 7.00)	0.071
Firmicutes/Bacteroidetes ratio	1.25 (1.20; 1.33)	1.20 (1.00; 1.30)	1.30 (1.20; 1.65)	1.20 (0.85; 1.60)	0.713
Butyrate production (µmol/g)	11.6 (9.28; 14.03)	11.5 (9.20; 14.40)	13.10 (11.15; 17.95)	12.30 (9.60; 16.15)	0.522
Lactate production (µmol/g)	1.40 (1.00; 2.43)	0.40 (0.10; 0.70)	0.40 (0.10; 0.45)	0.40 (0.20; 1.05)	0.014
Actetate/Propionate production (µmol/g)	33.20 (27.00; 37.60)	39.60 (32.80; 42.20)	15.60 (12.90; 28.00)	24.10 (23.40; 40.10)	0.039
LPS-positive bacteria (%)	2.00 (1.56; 4.38)	2.78 (1.18; 4.68)	3.20 (1.49; 4.42)	1.91 (1.08; 3.77)	0.828

HC = healthy control; AG = anxiety group; AI = autoimmunity group, IBS = irritable bowel syndrome group.

## Data Availability

The anonymized dataset supporting the findings of this study, including relative abundance values at phylum and genus level, short-chain fatty acid measurements, and clinical and anthropometric parameters for all 59 participants, is available as Supplementary Materials (Supplementary Table S3). All scripts and code used for data processing, statistical analysis, and figure generation are publicly available at: www.github.com/RaresCandrea/Candrea_et_al_2026_gut_microbiota—accessed on 22 April 2026.

## Supplementary Materials

The following supporting information can be downloaded at: https://www.mdpi.com/article/10.3390/biomedicines14051005/s1. Figure S1: Spearman correlation heatmap between gut microbiota, short-chain fatty acids, and clinical parameters, Table S1: Differential abundance analysis across study groups using LinDA (Linear Models for Differential Abundance), Table S2: Spearman correlation analysis between gut microbiota relative abundances and clinical parameters, including short-chain fatty acids and metabolic markers, Table S3: Anonymized dataset used for microbiome, short-chain fatty acid, and clinical parameter analyses.

## Abbreviations

The following abbreviations are used in this manuscript:

AG	Anxiety disorders group
AI	Autoimmune diseases group
BMI	Body mass index
CLR	Centered Log-Ratio
FDR	False Discovery Rate
HC	Healthy controls
HDL	High-density lipoprotein cholesterol
HS-GC–MS	Headspace gas chromatography–mass spectrometry
IBS	Irritable bowel syndrome
LDL	Low-density lipoprotein
LinDA	Linear models for differential abundance analysis
NGS	Next-generation sequencing
PCoA	Principal Coordinates Analysis
PERMANOVA	Permutational Multivariate Analysis of Variance
SCFA	Short-Chain Fatty Acids
