# Supplementary material for: Comparative Analysis of Gut Microbiota Patterns in Irritable Bowel Syndrome, Anxiety, and Autoimmune Disorders
Source: Biomedicines. 2026 Apr 28;14(5):1005. doi: 10.3390/biomedicines14051005 (PMC13205036; doi:10.3390/biomedicines14051005)
Supplement: Supplementary file 1 [file biomedicines-14-01005-s001.zip › Supplementary materials.pdf]

Supplementary Materials

Comparative Analysis of Gut Microbiota Patterns in Irritable Bowel Syndrome, Anxiety, and Autoimmune Disorders

Adelin-Rareș Candrea [1], Laura Ioana Gavrilaș [2,3,*], Andrei Mocan [1], Adriana Rusu [2,4] and Gianina Crișan [1]

[1] Department of Pharmaceutical Botany, Faculty of Pharmacy, "Iuliu Hațieganu" University of Medicine and Pharmacy, 23 Gheorghe Marinescu Street, 400337 Cluj-Napoca, Romania; adelin.rare.candrea@elearn.umfcluj.ro
[2] 2nd Department, Faculty of Nursing and Health Sciences, "Iuliu Hațieganu" University of Medicine and Pharmacy, 23 Gheorghe Marinescu Street, 400337 Cluj-Napoca, Romania; laura.gavrilas@umfcluj.ro
[3] Department of Development, Innovation and Research, Romanian Dietitians Association, 6 Dionisie Roman Street, 400394, Cluj-Napoca, Romania
[4] Diabetes Center, Emergency Clinical County Hospital Cluj, 400006 Cluj-Napoca, Romania
* Correspondence: laura.gavrilas@umfcluj.ro

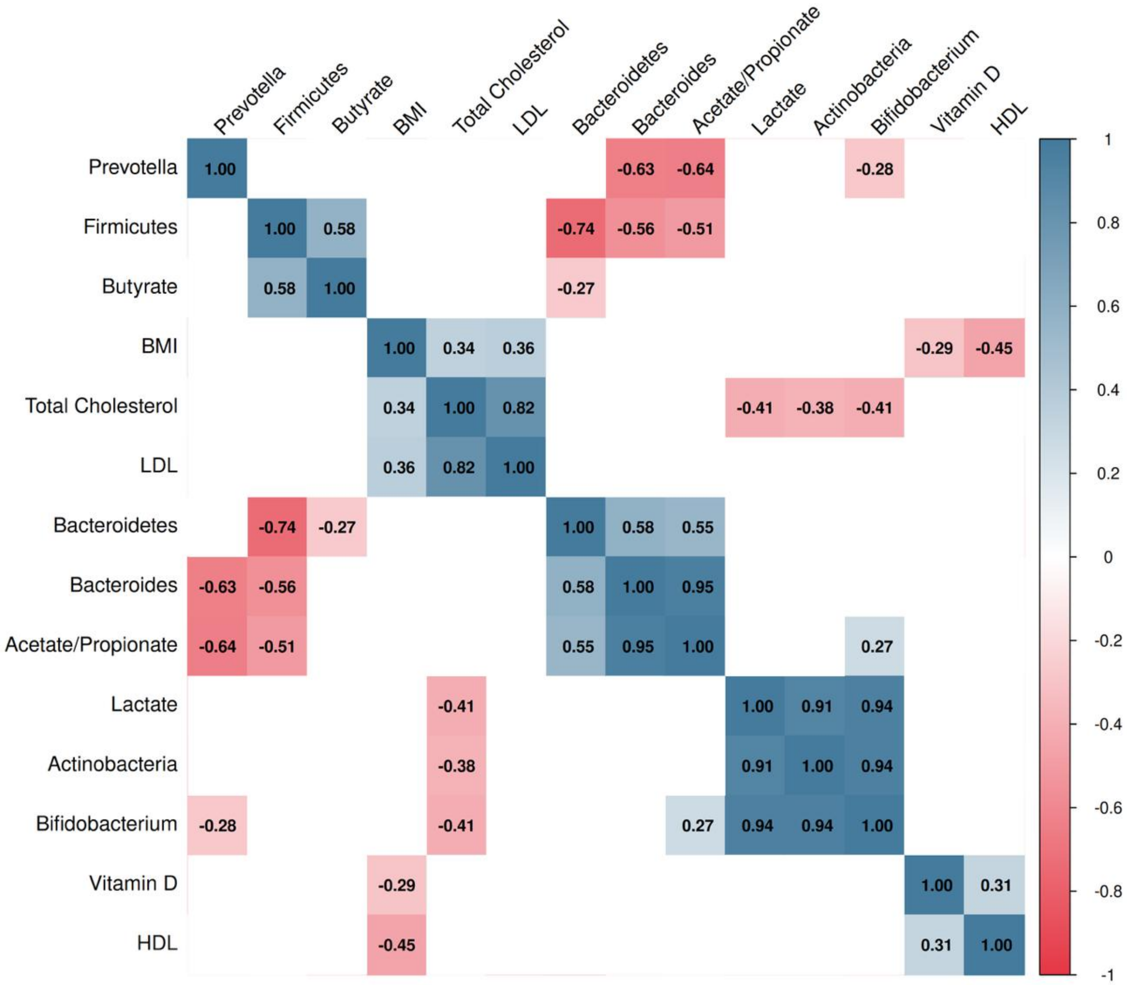

Figure S1. Spearman correlation heatmap between gut microbiota, short-chain fatty acids, and clinical parameters. Correlation coefficients (Spearman's rho) are shown. Positive correlations are indicated in blue and negative correlations in red. Only statistically significant correlations ($p < 0.05$) are displayed.

Table S1. Differential abundance analysis across study groups using LinDA (Linear Models for Differential Abundance), including log2 fold change, p-values, and false discovery rate (FDR)-adjusted p-values. Differential abundance analysis was performed using LinDA. Log2FoldChange values represent the direction and magnitude of change between groups (positive values indicate higher abundance in the first group of the comparison). P-values were adjusted for multiple testing using the false discovery rate (FDR) method. Statistically significant results (FDR-adjusted $p < 0.05$) are highlighted in bold/red. Only Actinobacteria showed significant differences after FDR correction.

Taxon	Comparison	log2FoldChange	pvalue	padj	Level
Firmicutes	AG_vs_HC	0.14403064	0.72879857	0.9012063	Phylum
Bacteroidetes	AG_vs_HC	0.20770959	0.66356448	0.9012063	Phylum
Proteobacteria	AG_vs_HC	-0.0812597	0.9012063	0.9012063	Phylum
Actinobacteria	AG_vs_HC	-1.8965065	0.0054544	0.0490896	Phylum
Verrucomicrobia	AG_vs_HC	2.56798271	0.15683076	0.61209815	Phylum
Fusobacteria	AG_vs_HC	0.99331089	0.27204362	0.61209815	Phylum
Cyanobacteria	AG_vs_HC	-0.2191712	0.83781161	0.9012063	Phylum
Euryarchaeota	AG_vs_HC	-0.2545468	0.70475544	0.9012063	Phylum
Tenericutes	AG_vs_HC	1.61958308	0.21048281	0.61209815	Phylum
Firmicutes	AI_vs_HC	0.35561692	0.40894373	0.73423754	Phylum
Bacteroidetes	AI_vs_HC	0.08859162	0.8575036	0.96469155	Phylum
Proteobacteria	AI_vs_HC	-0.0289431	0.96589128	0.96589128	Phylum
Actinobacteria	AI_vs_HC	-1.7051355	0.0148316	0.13348442	Phylum
Verrucomicrobia	AI_vs_HC	1.38952191	0.45578231	0.73423754	Phylum
Fusobacteria	AI_vs_HC	0.90216432	0.33407267	0.73423754	Phylum
Cyanobacteria	AI_vs_HC	-0.5827101	0.59907481	0.77023905	Phylum
Euryarchaeota	AI_vs_HC	-0.4808307	0.4894917	0.73423754	Phylum
Tenericutes	AI_vs_HC	2.73730809	0.04307085	0.19381883	Phylum
Firmicutes	IBS_vs_HC	0.5131601	0.17132364	0.29614915	Phylum
Bacteroidetes	IBS_vs_HC	0.55505979	0.19743277	0.29614915	Phylum
Proteobacteria	IBS_vs_HC	0.05433128	0.9261762	0.9261762	Phylum
Actinobacteria	IBS_vs_HC	-0.8292607	0.16351252	0.29614915	Phylum

Verrucomicrobia	IBS_vs_HC	2.71108716	0.09636804	0.29614915	Phylum
Fusobacteria	IBS_vs_HC	1.37361869	0.09239219	0.29614915	Phylum
Cyanobacteria	IBS_vs_HC	-0.2935241	0.75964444	0.85459999	Phylum
Euryarchaeota	IBS_vs_HC	-0.6439609	0.28677848	0.36871519	Phylum
Tenericutes	IBS_vs_HC	1.8360231	0.11456904	0.29614915	Phylum
Firmicutes	AI_vs_AG	0.26634111	0.48263511	0.96459875	Phylum
Bacteroidetes	AI_vs_AG	-0.0643631	0.88237135	0.96459875	Phylum
Proteobacteria	AI_vs_AG	0.10707146	0.85762318	0.96459875	Phylum
Actinobacteria	AI_vs_AG	0.24612576	0.68204653	0.96459875	Phylum
Verrucomicrobia	AI_vs_AG	-1.123706	0.4937442	0.96459875	Phylum
Fusobacteria	AI_vs_AG	-0.0363917	0.96459875	0.96459875	Phylum
Cyanobacteria	AI_vs_AG	-0.3087841	0.75182123	0.96459875	Phylum
Euryarchaeota	AI_vs_AG	-0.1715291	0.77936276	0.96459875	Phylum
Tenericutes	AI_vs_AG	1.17247984	0.31876902	0.96459875	Phylum
Firmicutes	HC_vs_AG	-0.1440306	0.72879857	0.9012063	Phylum
Bacteroidetes	HC_vs_AG	-0.2077096	0.66356448	0.9012063	Phylum
Proteobacteria	HC_vs_AG	0.08125974	0.9012063	0.9012063	Phylum
Actinobacteria	HC_vs_AG	1.89650646	0.0054544	0.0490896	Phylum
Verrucomicrobia	HC_vs_AG	-2.5679827	0.15683076	0.61209815	Phylum
Fusobacteria	HC_vs_AG	-0.9933109	0.27204362	0.61209815	Phylum
Cyanobacteria	HC_vs_AG	0.21917121	0.83781161	0.9012063	Phylum
Euryarchaeota	HC_vs_AG	0.25454681	0.70475544	0.9012063	Phylum
Tenericutes	HC_vs_AG	-1.6195831	0.21048281	0.61209815	Phylum
Firmicutes	IBS_vs_AG	0.06280001	0.84045668	0.9257613	Phylum
Bacteroidetes	IBS_vs_AG	0.04102074	0.90888431	0.9257613	Phylum
Proteobacteria	IBS_vs_AG	-0.1707384	0.72856879	0.9257613	Phylum
Actinobacteria	IBS_vs_AG	0.76091632	0.12803246	0.77134219	Phylum
Verrucomicrobia	IBS_vs_AG	-0.163225	0.90377898	0.9257613	Phylum
Fusobacteria	IBS_vs_AG	0.07397834	0.91281591	0.9257613	Phylum
Cyanobacteria	IBS_vs_AG	-0.3806824	0.63630867	0.9257613	Phylum
Euryarchaeota	IBS_vs_AG	-0.6957436	0.17140937	0.77134219	Phylum
Tenericutes	IBS_vs_AG	-0.0898894	0.9257613	0.9257613	Phylum
Firmicutes	AG_vs_AI	-0.2663411	0.48263511	0.96459875	Phylum
Bacteroidetes	AG_vs_AI	0.06436314	0.88237135	0.96459875	Phylum
Proteobacteria	AG_vs_AI	-0.1070715	0.85762318	0.96459875	Phylum
Actinobacteria	AG_vs_AI	-0.2461258	0.68204653	0.96459875	Phylum
Verrucomicrobia	AG_vs_AI	1.12370597	0.4937442	0.96459875	Phylum
Fusobacteria	AG_vs_AI	0.03639173	0.96459875	0.96459875	Phylum
Cyanobacteria	AG_vs_AI	0.3087841	0.75182123	0.96459875	Phylum
Euryarchaeota	AG_vs_AI	0.1715291	0.77936276	0.96459875	Phylum

Tenericutes	AG_vs_AI	-1.1724798	0.31876902	0.96459875	Phylum
Firmicutes	HC_vs_AI	-0.3556169	0.40894373	0.73423754	Phylum
Bacteroidetes	HC_vs_AI	-0.0885916	0.8575036	0.96469155	Phylum
Proteobacteria	HC_vs_AI	0.02894311	0.96589128	0.96589128	Phylum
Actinobacteria	HC_vs_AI	1.70513553	0.0148316	0.13348442	Phylum
Verrucomicrobia	HC_vs_AI	-1.3895219	0.45578231	0.73423754	Phylum
Fusobacteria	HC_vs_AI	-0.9021643	0.33407267	0.73423754	Phylum
Cyanobacteria	HC_vs_AI	0.58271015	0.59907481	0.77023905	Phylum
Euryarchaeota	HC_vs_AI	0.48083074	0.4894917	0.73423754	Phylum
Tenericutes	HC_vs_AI	-2.7373081	0.04307085	0.19381883	Phylum
Firmicutes	IBS_vs_AI	-0.1287935	0.69695109	0.89552331	Phylum
Bacteroidetes	IBS_vs_AI	0.1801315	0.63561637	0.89552331	Phylum
Proteobacteria	IBS_vs_AI	-0.2030623	0.69693404	0.89552331	Phylum
Actinobacteria	IBS_vs_AI	0.58953818	0.2634404	0.89552331	Phylum
Verrucomicrobia	IBS_vs_AI	1.03522857	0.47034202	0.89552331	Phylum
Fusobacteria	IBS_vs_AI	0.18511769	0.79602072	0.89552331	Phylum
Cyanobacteria	IBS_vs_AI	0.00284936	0.9973321	0.9973321	Phylum
Euryarchaeota	IBS_vs_AI	-0.4494669	0.40182861	0.89552331	Phylum
Tenericutes	IBS_vs_AI	-1.1876217	0.24816881	0.89552331	Phylum
Firmicutes	AG_vs_IBS	-0.0628	0.84045668	0.9257613	Phylum
Bacteroidetes	AG_vs_IBS	-0.0410207	0.90888431	0.9257613	Phylum
Proteobacteria	AG_vs_IBS	0.17073845	0.72856879	0.9257613	Phylum
Actinobacteria	AG_vs_IBS	-0.7609163	0.12803246	0.77134219	Phylum
Verrucomicrobia	AG_vs_IBS	0.16322501	0.90377898	0.9257613	Phylum
Fusobacteria	AG_vs_IBS	-0.0739783	0.91281591	0.9257613	Phylum
Cyanobacteria	AG_vs_IBS	0.38068236	0.63630867	0.9257613	Phylum
Euryarchaeota	AG_vs_IBS	0.69574357	0.17140937	0.77134219	Phylum
Tenericutes	AG_vs_IBS	0.08988944	0.9257613	0.9257613	Phylum
Firmicutes	AI_vs_IBS	0.12879349	0.69695109	0.89552331	Phylum
Bacteroidetes	AI_vs_IBS	-0.1801315	0.63561637	0.89552331	Phylum
Proteobacteria	AI_vs_IBS	0.20306229	0.69693404	0.89552331	Phylum
Actinobacteria	AI_vs_IBS	-0.5895382	0.2634404	0.89552331	Phylum
Verrucomicrobia	AI_vs_IBS	-1.0352286	0.47034202	0.89552331	Phylum
Fusobacteria	AI_vs_IBS	-0.1851177	0.79602072	0.89552331	Phylum
Cyanobacteria	AI_vs_IBS	-0.0028494	0.9973321	0.9973321	Phylum
Euryarchaeota	AI_vs_IBS	0.44946686	0.40182861	0.89552331	Phylum
Tenericutes	AI_vs_IBS	1.18762167	0.24816881	0.89552331	Phylum
Firmicutes	HC_vs_IBS	-0.5131601	0.17132364	0.29614915	Phylum
Bacteroidetes	HC_vs_IBS	-0.5550598	0.19743277	0.29614915	Phylum
Proteobacteria	HC_vs_IBS	-0.0543313	0.9261762	0.9261762	Phylum

Actinobacteria	HC_vs_IBS	0.82926068	0.16351252	0.29614915	Phylum
Verrucomicrobia	HC_vs_IBS	-2.7110872	0.09636804	0.29614915	Phylum
Fusobacteria	HC_vs_IBS	-1.3736187	0.09239219	0.29614915	Phylum
Cyanobacteria	HC_vs_IBS	0.29352411	0.75964444	0.85459999	Phylum
Euryarchaeota	HC_vs_IBS	0.64396092	0.28677848	0.36871519	Phylum
Tenericutes	HC_vs_IBS	-1.8360231	0.11456904	0.29614915	Phylum
Prevotella_spp	AG_vs_HC	-0.5037876	0.83344223	0.96833124	Genus
Desulfobacter_spp	AG_vs_HC	-0.0900424	0.91002609	0.96833124	Genus
Desulfovibrio_spp	AG_vs_HC	0.32034974	0.80464336	0.96833124	Genus
Oscillibacter_spp	AG_vs_HC	0.26625848	0.78747599	0.96833124	Genus
Alistipes_spp	AG_vs_HC	-0.7599285	0.47980131	0.96833124	Genus
Methanobrevibacter_spp	AG_vs_HC	-0.6812697	0.23097369	0.7919098	Genus
Methanobacteria	AG_vs_HC	-0.8192755	0.18508398	0.74033593	Genus
Citrobacter_spp	AG_vs_HC	-1.9649412	0.151637	0.72785758	Genus
Enterobacter_spp	AG_vs_HC	0.25242693	0.87356252	0.96833124	Genus
Escherichia_spp	AG_vs_HC	-1.2976318	0.32164784	0.85772757	Genus
Klebsiella_spp	AG_vs_HC	-0.0718629	0.96378383	0.96833124	Genus
Providencia_spp	AG_vs_HC	0.12351646	0.71962218	0.96833124	Genus
Pseudomonas_spp	AG_vs_HC	0.04546365	0.96833124	0.96833124	Genus
Serratia_spp	AG_vs_HC	-0.0366077	0.91416249	0.96833124	Genus
Sutterella_spp	AG_vs_HC	0.46492499	0.76055837	0.96833124	Genus
Enterococcus_spp	AG_vs_HC	-0.9017405	0.46575599	0.96833124	Genus
Ruminococcus_spp	AG_vs_HC	-0.2829285	0.55970774	0.96833124	Genus
Eubacterium_spp	AG_vs_HC	-1.8595556	0.09470949	0.72785758	Genus
Bacteroides_spp	AG_vs_HC	0.23615215	0.74916877	0.96833124	Genus
Dorea_spp	AG_vs_HC	-0.7015687	0.28580355	0.85741064	Genus
Bifidobacterium_spp	AG_vs_HC	-3.7963047	0.00608451	0.14602833	Genus
Lactobacillus_spp	AG_vs_HC	-1.6604547	0.12133342	0.72785758	Genus
Clostridium_spp	AG_vs_HC	-0.0943553	0.85372196	0.96833124	Genus
Streptococcus_spp	AG_vs_HC	-1.3983834	0.11115361	0.72785758	Genus
Prevotella_spp	AI_vs_HC	5.10314925	0.04318156	0.35076253	Genus
Desulfobacter_spp	AI_vs_HC	0.37125755	0.65256448	0.87008598	Genus
Desulfovibrio_spp	AI_vs_HC	2.83151192	0.03814284	0.35076253	Genus
Oscillibacter_spp	AI_vs_HC	1.85713802	0.07307553	0.35076253	Genus
Alistipes_spp	AI_vs_HC	0.00845628	0.99391891	0.99391891	Genus
Methanobrevibacter_spp	AI_vs_HC	-0.0481036	0.93438306	0.97500841	Genus
Methanobacteria	AI_vs_HC	-0.1316098	0.8356131	0.97500841	Genus
Citrobacter_spp	AI_vs_HC	-2.0481664	0.14843852	0.40008145	Genus
Enterobacter_spp	AI_vs_HC	-0.9286544	0.5718017	0.85640313	Genus
Escherichia_spp	AI_vs_HC	-1.2731137	0.34683247	0.7567254	Genus

Klebsiella_spp	AI_vs_HC	-0.8436979	0.60661888	0.85640313	Genus
Providencia_spp	AI_vs_HC	0.53419282	0.13700251	0.40008145	Genus
Pseudomonas_spp	AI_vs_HC	0.14452668	0.90286343	0.97500841	Genus
Serratia_spp	AI_vs_HC	0.67828032	0.05747666	0.35076253	Genus
Sutterella_spp	AI_vs_HC	2.3983297	0.13227924	0.40008145	Genus
Enterococcus_spp	AI_vs_HC	-0.8432561	0.50930207	0.81488332	Genus
Ruminococcus_spp	AI_vs_HC	0.72776472	0.15003054	0.40008145	Genus
Eubacterium_spp	AI_vs_HC	0.77989396	0.4933145	0.81488332	Genus
Bacteroides_spp	AI_vs_HC	-0.2131187	0.78018446	0.97500841	Genus
Dorea_spp	AI_vs_HC	-0.0841018	0.90101497	0.97500841	Genus
Bifidobacterium_spp	AI_vs_HC	-1.8331576	0.18819269	0.45166246	Genus
Lactobacillus_spp	AI_vs_HC	-0.8255691	0.45252833	0.81488332	Genus
Clostridium_spp	AI_vs_HC	0.98018915	0.06809863	0.35076253	Genus
Streptococcus_spp	AI_vs_HC	-0.6995021	0.43685618	0.81488332	Genus
Prevotella_spp	IBS_vs_HC	1.169529	0.58621294	0.99921635	Genus
Desulfobacter_spp	IBS_vs_HC	-0.0231566	0.97411786	0.99921635	Genus
Desulfovibrio_spp	IBS_vs_HC	0.86918868	0.45479073	0.99921635	Genus
Oscillibacter_spp	IBS_vs_HC	0.30387475	0.73130359	0.99921635	Genus
Alistipes_spp	IBS_vs_HC	-1.1060669	0.25268701	0.99921635	Genus
Methanobrevibacter_spp	IBS_vs_HC	-1.2149873	0.01925592	0.15404736	Genus
Methanobacteria	IBS_vs_HC	-1.3894026	0.01391318	0.15404736	Genus
Citrobacter_spp	IBS_vs_HC	-0.789539	0.51700299	0.99921635	Genus
Enterobacter_spp	IBS_vs_HC	0.08899444	0.95005668	0.99921635	Genus
Escherichia_spp	IBS_vs_HC	-1.6410503	0.16362144	0.98172865	Genus
Klebsiella_spp	IBS_vs_HC	0.0490998	0.97237341	0.99921635	Genus
Providencia_spp	IBS_vs_HC	-0.0183438	0.9525176	0.99921635	Genus
Pseudomonas_spp	IBS_vs_HC	-0.7885175	0.44331701	0.99921635	Genus
Serratia_spp	IBS_vs_HC	0.00029879	0.99921635	0.99921635	Genus
Sutterella_spp	IBS_vs_HC	-0.0965936	0.94363043	0.99921635	Genus
Enterococcus_spp	IBS_vs_HC	-0.6669762	0.54669326	0.99921635	Genus
Ruminococcus_spp	IBS_vs_HC	-0.0453335	0.91678382	0.99921635	Genus
Eubacterium_spp	IBS_vs_HC	-0.6946069	0.48130527	0.99921635	Genus
Bacteroides_spp	IBS_vs_HC	-0.2043907	0.75737507	0.99921635	Genus
Dorea_spp	IBS_vs_HC	-0.6195223	0.29264776	0.99921635	Genus
Bifidobacterium_spp	IBS_vs_HC	-2.9192975	0.01751962	0.15404736	Genus
Lactobacillus_spp	IBS_vs_HC	0.18947101	0.8418794	0.99921635	Genus
Clostridium_spp	IBS_vs_HC	0.20514133	0.65477932	0.99921635	Genus
Streptococcus_spp	IBS_vs_HC	-0.622624	0.42444043	0.99921635	Genus
Prevotella_spp	AI_vs_AG	4.99047125	0.02552563	0.56771206	Genus
Desulfobacter_spp	AI_vs_AG	-0.1551656	0.83088309	0.99865983	Genus

Desulfovibrio_spp	AI_vs_AG	1.89469659	0.11260562	0.70448849	Genus
Oscillibacter_spp	AI_vs_AG	0.97441394	0.28157209	0.81528937	Genus
Alistipes_spp	AI_vs_AG	0.15191922	0.87659691	0.99865983	Genus
Methanobrevibacter_spp	AI_vs_AG	0.01670059	0.97413608	0.99865983	Genus
Methanobacteria	AI_vs_AG	0.07120004	0.89866822	0.99865983	Genus
Citrobacter_spp	AI_vs_AG	-0.6996908	0.57241739	0.93085795	Genus
Enterobacter_spp	AI_vs_AG	-1.7975469	0.21703065	0.81528937	Genus
Escherichia_spp	AI_vs_AG	-0.5919475	0.61878356	0.93085795	Genus
Klebsiella_spp	AI_vs_AG	-1.3883006	0.3380008	0.81528937	Genus
Providencia_spp	AI_vs_AG	-0.2057892	0.51240489	0.93085795	Genus
Pseudomonas_spp	AI_vs_AG	-0.5174026	0.62057197	0.93085795	Genus
Serratia_spp	AI_vs_AG	0.0984224	0.75067945	0.99865983	Genus
Sutterella_spp	AI_vs_AG	1.31693911	0.34548212	0.81528937	Genus
Enterococcus_spp	AI_vs_AG	-0.5579812	0.62009696	0.93085795	Genus
Ruminococcus_spp	AI_vs_AG	0.39422759	0.37367429	0.81528937	Genus
Eubacterium_spp	AI_vs_AG	2.02298398	0.04730934	0.56771206	Genus
Bacteroides_spp	AI_vs_AG	-1.0657365	0.11741475	0.70448849	Genus
Dorea_spp	AI_vs_AG	0.00100126	0.99865983	0.99865983	Genus
Bifidobacterium_spp	AI_vs_AG	1.34668149	0.27171399	0.81528937	Genus
Lactobacillus_spp	AI_vs_AG	0.21842	0.82123798	0.99865983	Genus
Clostridium_spp	AI_vs_AG	0.4580789	0.32824361	0.81528937	Genus
Streptococcus_spp	AI_vs_AG	0.08241567	0.91702196	0.99865983	Genus
Prevotella_spp	HC_vs_AG	0.50378759	0.83344223	0.96833124	Genus
Desulfobacter_spp	HC_vs_AG	0.09004243	0.91002609	0.96833124	Genus
Desulfovibrio_spp	HC_vs_AG	-0.3203497	0.80464336	0.96833124	Genus
Oscillibacter_spp	HC_vs_AG	-0.2662585	0.78747599	0.96833124	Genus
Alistipes_spp	HC_vs_AG	0.75992853	0.47980131	0.96833124	Genus
Methanobrevibacter_spp	HC_vs_AG	0.68126974	0.23097369	0.7919098	Genus
Methanobacteria	HC_vs_AG	0.81927546	0.18508398	0.74033593	Genus
Citrobacter_spp	HC_vs_AG	1.96494116	0.151637	0.72785758	Genus
Enterobacter_spp	HC_vs_AG	-0.2524269	0.87356252	0.96833124	Genus
Escherichia_spp	HC_vs_AG	1.29763179	0.32164784	0.85772757	Genus
Klebsiella_spp	HC_vs_AG	0.07186293	0.96378383	0.96833124	Genus
Providencia_spp	HC_vs_AG	-0.1235165	0.71962218	0.96833124	Genus
Pseudomonas_spp	HC_vs_AG	-0.0454636	0.96833124	0.96833124	Genus
Serratia_spp	HC_vs_AG	0.03660768	0.91416249	0.96833124	Genus
Sutterella_spp	HC_vs_AG	-0.464925	0.76055837	0.96833124	Genus
Enterococcus_spp	HC_vs_AG	0.90174049	0.46575599	0.96833124	Genus
Ruminococcus_spp	HC_vs_AG	0.28292847	0.55970774	0.96833124	Genus
Eubacterium_spp	HC_vs_AG	1.85955563	0.09470949	0.72785758	Genus

Bacteroides_spp	HC_vs_AG	-0.2361522	0.74916877	0.96833124	Genus
Dorea_spp	HC_vs_AG	0.70156869	0.28580355	0.85741064	Genus
Bifidobacterium_spp	HC_vs_AG	3.79630471	0.00608451	0.14602833	Genus
Lactobacillus_spp	HC_vs_AG	1.66045465	0.12133342	0.72785758	Genus
Clostridium_spp	HC_vs_AG	0.09435534	0.85372196	0.96833124	Genus
Streptococcus_spp	HC_vs_AG	1.39838336	0.11115361	0.72785758	Genus
Prevotella_spp	IBS_vs_AG	1.72324299	0.34023947	0.87637121	Genus
Desulfobacter_spp	IBS_vs_AG	0.11681226	0.84529699	0.92411049	Genus
Desulfovibrio_spp	IBS_vs_AG	0.59876533	0.53888923	0.92411049	Genus
Oscillibacter_spp	IBS_vs_AG	0.08754267	0.90605239	0.92411049	Genus
Alistipes_spp	IBS_vs_AG	-0.2962119	0.71343421	0.92411049	Genus
Methanobrevibacter_spp	IBS_vs_AG	-0.4837911	0.25718028	0.87637121	Genus
Methanobacteria	IBS_vs_AG	-0.5202008	0.26157937	0.87637121	Genus
Citrobacter_spp	IBS_vs_AG	1.22532856	0.23263133	0.87637121	Genus
Enterobacter_spp	IBS_vs_AG	-0.1135061	0.92411049	0.92411049	Genus
Escherichia_spp	IBS_vs_AG	-0.2934921	0.76447952	0.92411049	Genus
Klebsiella_spp	IBS_vs_AG	0.17088914	0.88572588	0.92411049	Genus
Providencia_spp	IBS_vs_AG	-0.0919338	0.72210299	0.92411049	Genus
Pseudomonas_spp	IBS_vs_AG	-0.7840548	0.36390125	0.87637121	Genus
Serratia_spp	IBS_vs_AG	0.08683287	0.73372686	0.92411049	Genus
Sutterella_spp	IBS_vs_AG	-0.5115922	0.65548467	0.92411049	Genus
Enterococcus_spp	IBS_vs_AG	0.28469071	0.7587314	0.92411049	Genus
Ruminococcus_spp	IBS_vs_AG	0.2875214	0.4306983	0.92411049	Genus
Eubacterium_spp	IBS_vs_AG	1.21487514	0.14492229	0.87637121	Genus
Bacteroides_spp	IBS_vs_AG	-0.3906164	0.482226	0.92411049	Genus
Dorea_spp	IBS_vs_AG	0.13197279	0.78825305	0.92411049	Genus
Bifidobacterium_spp	IBS_vs_AG	0.92693363	0.35776455	0.87637121	Genus
Lactobacillus_spp	IBS_vs_AG	1.89985206	0.01997808	0.47947401	Genus
Clostridium_spp	IBS_vs_AG	0.34942307	0.36515467	0.87637121	Genus
Streptococcus_spp	IBS_vs_AG	0.82568573	0.20853635	0.87637121	Genus
Prevotella_spp	AG_vs_AI	-4.9904712	0.02552563	0.56771206	Genus
Desulfobacter_spp	AG_vs_AI	0.15516562	0.83088309	0.99865983	Genus
Desulfovibrio_spp	AG_vs_AI	-1.8946966	0.11260562	0.70448849	Genus
Oscillibacter_spp	AG_vs_AI	-0.9744139	0.28157209	0.81528937	Genus
Alistipes_spp	AG_vs_AI	-0.1519192	0.87659691	0.99865983	Genus
Methanobrevibacter_spp	AG_vs_AI	-0.0167006	0.97413608	0.99865983	Genus
Methanobacteria	AG_vs_AI	-0.0712	0.89866822	0.99865983	Genus
Citrobacter_spp	AG_vs_AI	0.69969085	0.57241739	0.93085795	Genus
Enterobacter_spp	AG_vs_AI	1.79754689	0.21703065	0.81528937	Genus
Escherichia_spp	AG_vs_AI	0.59194754	0.61878356	0.93085795	Genus

Klebsiella_spp	AG_vs_AI	1.38830056	0.3380008	0.81528937	Genus
Providencia_spp	AG_vs_AI	0.20578924	0.51240489	0.93085795	Genus
Pseudomonas_spp	AG_vs_AI	0.51740256	0.62057197	0.93085795	Genus
Serratia_spp	AG_vs_AI	-0.0984224	0.75067945	0.99865983	Genus
Sutterella_spp	AG_vs_AI	-1.3169391	0.34548212	0.81528937	Genus
Enterococcus_spp	AG_vs_AI	0.5579812	0.62009696	0.93085795	Genus
Ruminococcus_spp	AG_vs_AI	-0.3942276	0.37367429	0.81528937	Genus
Eubacterium_spp	AG_vs_AI	-2.022984	0.04730934	0.56771206	Genus
Bacteroides_spp	AG_vs_AI	1.06573647	0.11741475	0.70448849	Genus
Dorea_spp	AG_vs_AI	-0.0010013	0.99865983	0.99865983	Genus
Bifidobacterium_spp	AG_vs_AI	-1.3466815	0.27171399	0.81528937	Genus
Lactobacillus_spp	AG_vs_AI	-0.21842	0.82123798	0.99865983	Genus
Clostridium_spp	AG_vs_AI	-0.4580789	0.32824361	0.81528937	Genus
Streptococcus_spp	AG_vs_AI	-0.0824157	0.91702196	0.99865983	Genus
Prevotella_spp	HC_vs_AI	-5.1031493	0.04318156	0.35076253	Genus
Desulfobacter_spp	HC_vs_AI	-0.3712576	0.65256448	0.87008598	Genus
Desulfovibrio_spp	HC_vs_AI	-2.8315119	0.03814284	0.35076253	Genus
Oscillibacter_spp	HC_vs_AI	-1.857138	0.07307553	0.35076253	Genus
Alistipes_spp	HC_vs_AI	-0.0084563	0.99391891	0.99391891	Genus
Methanobrevibacter_spp	HC_vs_AI	0.04810355	0.93438306	0.97500841	Genus
Methanobacteria	HC_vs_AI	0.13160983	0.8356131	0.97500841	Genus
Citrobacter_spp	HC_vs_AI	2.04816641	0.14843852	0.40008145	Genus
Enterobacter_spp	HC_vs_AI	0.92865436	0.5718017	0.85640313	Genus
Escherichia_spp	HC_vs_AI	1.27311373	0.34683247	0.7567254	Genus
Klebsiella_spp	HC_vs_AI	0.8436979	0.60661888	0.85640313	Genus
Providencia_spp	HC_vs_AI	-0.5341928	0.13700251	0.40008145	Genus
Pseudomonas_spp	HC_vs_AI	-0.1445267	0.90286343	0.97500841	Genus
Serratia_spp	HC_vs_AI	-0.6782803	0.05747666	0.35076253	Genus
Sutterella_spp	HC_vs_AI	-2.3983297	0.13227924	0.40008145	Genus
Enterococcus_spp	HC_vs_AI	0.8432561	0.50930207	0.81488332	Genus
Ruminococcus_spp	HC_vs_AI	-0.7277647	0.15003054	0.40008145	Genus
Eubacterium_spp	HC_vs_AI	-0.779894	0.4933145	0.81488332	Genus
Bacteroides_spp	HC_vs_AI	0.21311872	0.78018446	0.97500841	Genus
Dorea_spp	HC_vs_AI	0.08410182	0.90101497	0.97500841	Genus
Bifidobacterium_spp	HC_vs_AI	1.83315762	0.18819269	0.45166246	Genus
Lactobacillus_spp	HC_vs_AI	0.82556906	0.45252833	0.81488332	Genus
Clostridium_spp	HC_vs_AI	-0.9801892	0.06809863	0.35076253	Genus
Streptococcus_spp	HC_vs_AI	0.69950209	0.43685618	0.81488332	Genus
Prevotella_spp	IBS_vs_AI	-3.117114	0.10622586	0.59764011	Genus
Desulfobacter_spp	IBS_vs_AI	0.42209217	0.50657397	0.75986096	Genus

Desulfovibrio_spp	IBS_vs_AI	-1.145817	0.26894718	0.67896144	Genus
Oscillibacter_spp	IBS_vs_AI	-0.736757	0.35045399	0.67896144	Genus
Alistipes_spp	IBS_vs_AI	-0.2980168	0.7272936	0.87275232	Genus
Methanobrevibacter_spp	IBS_vs_AI	-0.3503774	0.43722418	0.72474091	Genus
Methanobacteria	IBS_vs_AI	-0.4412865	0.36777078	0.67896144	Genus
Citrobacter_spp	IBS_vs_AI	2.0751337	0.05889741	0.59764011	Genus
Enterobacter_spp	IBS_vs_AI	1.83415509	0.15014745	0.59764011	Genus
Escherichia_spp	IBS_vs_AI	0.44856974	0.66576524	0.84096662	Genus
Klebsiella_spp	IBS_vs_AI	1.70930399	0.17844904	0.59764011	Genus
Providencia_spp	IBS_vs_AI	0.26396972	0.33693521	0.67896144	Genus
Pseudomonas_spp	IBS_vs_AI	-0.1165379	0.89827765	0.91893042	Genus
Serratia_spp	IBS_vs_AI	0.13852477	0.60877976	0.81170635	Genus
Sutterella_spp	IBS_vs_AI	-1.678417	0.17043816	0.59764011	Genus
Enterococcus_spp	IBS_vs_AI	0.99278621	0.31412263	0.67896144	Genus
Ruminococcus_spp	IBS_vs_AI	0.0434081	0.91035574	0.91893042	Genus
Eubacterium_spp	IBS_vs_AI	-0.6579945	0.45296307	0.72474091	Genus
Bacteroides_spp	IBS_vs_AI	0.82523432	0.16396582	0.59764011	Genus
Dorea_spp	IBS_vs_AI	0.28108582	0.58965843	0.81170635	Genus
Bifidobacterium_spp	IBS_vs_AI	-0.2696336	0.79998042	0.91426333	Genus
Lactobacillus_spp	IBS_vs_AI	1.83154636	0.03352124	0.59764011	Genus
Clostridium_spp	IBS_vs_AI	0.04145847	0.91893042	0.91893042	Genus
Streptococcus_spp	IBS_vs_AI	0.89338436	0.19921337	0.59764011	Genus
Prevotella_spp	AG_vs_IBS	-1.723243	0.34023947	0.87637121	Genus
Desulfobacter_spp	AG_vs_IBS	-0.1168123	0.84529699	0.92411049	Genus
Desulfovibrio_spp	AG_vs_IBS	-0.5987653	0.53888923	0.92411049	Genus
Oscillibacter_spp	AG_vs_IBS	-0.0875427	0.90605239	0.92411049	Genus
Alistipes_spp	AG_vs_IBS	0.29621192	0.71343421	0.92411049	Genus
Methanobrevibacter_spp	AG_vs_IBS	0.48379111	0.25718028	0.87637121	Genus
Methanobacteria	AG_vs_IBS	0.52020076	0.26157937	0.87637121	Genus
Citrobacter_spp	AG_vs_IBS	-1.2253286	0.23263133	0.87637121	Genus
Enterobacter_spp	AG_vs_IBS	0.1135061	0.92411049	0.92411049	Genus
Escherichia_spp	AG_vs_IBS	0.2934921	0.76447952	0.92411049	Genus
Klebsiella_spp	AG_vs_IBS	-0.1708891	0.88572588	0.92411049	Genus
Providencia_spp	AG_vs_IBS	0.09193381	0.72210299	0.92411049	Genus
Pseudomonas_spp	AG_vs_IBS	0.78405475	0.36390125	0.87637121	Genus
Serratia_spp	AG_vs_IBS	-0.0868329	0.73372686	0.92411049	Genus
Sutterella_spp	AG_vs_IBS	0.51159219	0.65548467	0.92411049	Genus
Enterococcus_spp	AG_vs_IBS	-0.2846907	0.7587314	0.92411049	Genus
Ruminococcus_spp	AG_vs_IBS	-0.2875214	0.4306983	0.92411049	Genus
Eubacterium_spp	AG_vs_IBS	-1.2148751	0.14492229	0.87637121	Genus

Bacteroides_spp	AG_vs_IBS	0.39061645	0.482226	0.92411049	Genus
Dorea_spp	AG_vs_IBS	-0.1319728	0.78825305	0.92411049	Genus
Bifidobacterium_spp	AG_vs_IBS	-0.9269336	0.35776455	0.87637121	Genus
Lactobacillus_spp	AG_vs_IBS	-1.8998521	0.01997808	0.47947401	Genus
Clostridium_spp	AG_vs_IBS	-0.3494231	0.36515467	0.87637121	Genus
Streptococcus_spp	AG_vs_IBS	-0.8256857	0.20853635	0.87637121	Genus
Prevotella_spp	AI_vs_IBS	3.11711396	0.10622586	0.59764011	Genus
Desulfobacter_spp	AI_vs_IBS	-0.4220922	0.50657397	0.75986096	Genus
Desulfovibrio_spp	AI_vs_IBS	1.14581695	0.26894718	0.67896144	Genus
Oscillibacter_spp	AI_vs_IBS	0.73675697	0.35045399	0.67896144	Genus
Alistipes_spp	AI_vs_IBS	0.29801684	0.7272936	0.87275232	Genus
Methanobrevibacter_spp	AI_vs_IBS	0.35037741	0.43722418	0.72474091	Genus
Methanobacteria	AI_vs_IBS	0.4412865	0.36777078	0.67896144	Genus
Citrobacter_spp	AI_vs_IBS	-2.0751337	0.05889741	0.59764011	Genus
Enterobacter_spp	AI_vs_IBS	-1.8341551	0.15014745	0.59764011	Genus
Escherichia_spp	AI_vs_IBS	-0.4485697	0.66576524	0.84096662	Genus
Klebsiella_spp	AI_vs_IBS	-1.709304	0.17844904	0.59764011	Genus
Providencia_spp	AI_vs_IBS	-0.2639697	0.33693521	0.67896144	Genus
Pseudomonas_spp	AI_vs_IBS	0.11653789	0.89827765	0.91893042	Genus
Serratia_spp	AI_vs_IBS	-0.1385248	0.60877976	0.81170635	Genus
Sutterella_spp	AI_vs_IBS	1.67841701	0.17043816	0.59764011	Genus
Enterococcus_spp	AI_vs_IBS	-0.9927862	0.31412263	0.67896144	Genus
Ruminococcus_spp	AI_vs_IBS	-0.0434081	0.91035574	0.91893042	Genus
Eubacterium_spp	AI_vs_IBS	0.65799455	0.45296307	0.72474091	Genus
Bacteroides_spp	AI_vs_IBS	-0.8252343	0.16396582	0.59764011	Genus
Dorea_spp	AI_vs_IBS	-0.2810858	0.58965843	0.81170635	Genus
Bifidobacterium_spp	AI_vs_IBS	0.26963357	0.79998042	0.91426333	Genus
Lactobacillus_spp	AI_vs_IBS	-1.8315464	0.03352124	0.59764011	Genus
Clostridium_spp	AI_vs_IBS	-0.0414585	0.91893042	0.91893042	Genus
Streptococcus_spp	AI_vs_IBS	-0.8933844	0.19921337	0.59764011	Genus
Prevotella_spp	HC_vs_IBS	-1.169529	0.58621294	0.99921635	Genus
Desulfobacter_spp	HC_vs_IBS	0.02315657	0.97411786	0.99921635	Genus
Desulfovibrio_spp	HC_vs_IBS	-0.8691887	0.45479073	0.99921635	Genus
Oscillibacter_spp	HC_vs_IBS	-0.3038748	0.73130359	0.99921635	Genus
Alistipes_spp	HC_vs_IBS	1.10606685	0.25268701	0.99921635	Genus
Methanobrevibacter_spp	HC_vs_IBS	1.21498725	0.01925592	0.15404736	Genus
Methanobacteria	HC_vs_IBS	1.38940262	0.01391318	0.15404736	Genus
Citrobacter_spp	HC_vs_IBS	0.789539	0.51700299	0.99921635	Genus
Enterobacter_spp	HC_vs_IBS	-0.0889944	0.95005668	0.99921635	Genus
Escherichia_spp	HC_vs_IBS	1.64105029	0.16362144	0.98172865	Genus

Klebsiella_spp	HC_vs_IBS	-0.0490998	0.97237341	0.99921635	Genus
Providencia_spp	HC_vs_IBS	0.01834375	0.9525176	0.99921635	Genus
Pseudomonas_spp	HC_vs_IBS	0.7885175	0.44331701	0.99921635	Genus
Serratia_spp	HC_vs_IBS	-0.0002988	0.99921635	0.99921635	Genus
Sutterella_spp	HC_vs_IBS	0.0965936	0.94363043	0.99921635	Genus
Enterococcus_spp	HC_vs_IBS	0.66697618	0.54669326	0.99921635	Genus
Ruminococcus_spp	HC_vs_IBS	0.04533347	0.91678382	0.99921635	Genus
Eubacterium_spp	HC_vs_IBS	0.69460688	0.48130527	0.99921635	Genus
Bacteroides_spp	HC_vs_IBS	0.20439069	0.75737507	0.99921635	Genus
Dorea_spp	HC_vs_IBS	0.6195223	0.29264776	0.99921635	Genus
Bifidobacterium_spp	HC_vs_IBS	2.91929748	0.01751962	0.15404736	Genus
Lactobacillus_spp	HC_vs_IBS	-0.189471	0.8418794	0.99921635	Genus
Clostridium_spp	HC_vs_IBS	-0.2051413	0.65477932	0.99921635	Genus
Streptococcus_spp	HC_vs_IBS	0.62262402	0.42444043	0.99921635	Genus
Akkermansia_muciniphila	AG_vs_HC	2.320912	0.22252158	0.9471177	Species
Prevotella_copri	AG_vs_HC	-0.5362817	0.83960776	0.965182	Species
Faecalibacterium_prausnitzii	AG_vs_HC	-0.9199887	0.47355885	0.9471177	Species
Bilophila_wadsworthia	AG_vs_HC	-0.0615078	0.95577381	0.965182	Species
Bifidobacterium_adolescentis	AG_vs_HC	-3.0264491	0.04501938	0.360155	Species
Bifidobacterium_dentium	AG_vs_HC	-0.1550143	0.89742373	0.965182	Species
Lactobacillus_plantarum	AG_vs_HC	0.09982605	0.84827858	0.965182	Species
Butyrivibrio_crossotus	AG_vs_HC	1.0758553	0.36897454	0.9471177	Species
Bacteroides_vulgatus	AG_vs_HC	-1.1560303	0.37315224	0.9471177	Species
Hafnia_alveii	AG_vs_HC	-0.6768401	0.53293985	0.94744862	Species
Clostridium_difficile	AG_vs_HC	-2.0669864	0.03829054	0.360155	Species
Clostridium_scindens	AG_vs_HC	1.3371821	0.28021153	0.9471177	Species
Fusobacterium_nucleatum	AG_vs_HC	0.40751835	0.46481495	0.9471177	Species
Oxalobacter_formigenes	AG_vs_HC	0.1909822	0.82971215	0.965182	Species
Anaerotruncus_colihominis	AG_vs_HC	-0.0270656	0.965182	0.965182	Species
Saccharomyces_cerevisiae	AG_vs_HC	0.11435206	0.92410943	0.965182	Species
Akkermansia_muciniphila	AI_vs_HC	0.12112927	0.95057174	0.95288327	Species
Prevotella_copri	AI_vs_HC	6.07820884	0.02992385	0.23939079	Species
Faecalibacterium_prausnitzii	AI_vs_HC	0.38529455	0.77117075	0.95288327	Species
Bilophila_wadsworthia	AI_vs_HC	0.06776401	0.95288327	0.95288327	Species
Bifidobacterium_adolescentis	AI_vs_HC	0.12810333	0.93338897	0.95288327	Species
Bifidobacterium_dentium	AI_vs_HC	-0.7991869	0.52114059	0.95288327	Species
Lactobacillus_plantarum	AI_vs_HC	0.20501386	0.70410852	0.95288327	Species
Butyrivibrio_crossotus	AI_vs_HC	1.70569501	0.17047817	0.82351873	Species
Bacteroides_vulgatus	AI_vs_HC	-0.8206859	0.54014467	0.95288327	Species

Hafnia_alveii	AI_vs_HC	-2.6058436	0.0231496	0.23939079	Species
Clostridium_difficile	AI_vs_HC	-0.3962234	0.69546132	0.95288327	Species
Clostridium_scindens	AI_vs_HC	-0.2885345	0.82082108	0.95288327	Species
Fusobacterium_nucleatum	AI_vs_HC	-0.2008651	0.72703317	0.95288327	Species
Oxalobacter_formigenes	AI_vs_HC	-0.7111646	0.43979573	0.95288327	Species
Anaerotruncus_colihominis	AI_vs_HC	-0.8170107	0.20587968	0.82351873	Species
Saccharomyces_cerevisiae	AI_vs_HC	0.13671197	0.91230376	0.95288327	Species
Akkermansia_muciniphila	IBS_vs_HC	2.72963771	0.11098137	0.53801561	Species
Prevotella_copri	IBS_vs_HC	2.83215529	0.2357253	0.6286008	Species
Faecalibacterium_prausnitzi i	IBS_vs_HC	0.01252846	0.9912865	0.9912865	Species
Bilophila_wadsworthia	IBS_vs_HC	1.3813043	0.16812988	0.53801561	Species
Bifidobacterium_adolescentis	IBS_vs_HC	-0.5653715	0.67049547	0.9912865	Species
Bifidobacterium_dentium	IBS_vs_HC	0.05994936	0.95561361	0.9912865	Species
Lactobacillus_plantarum	IBS_vs_HC	1.16292163	0.01544231	0.24707698	Species
Butyrivibrio_crossotus	IBS_vs_HC	0.49327844	0.64474127	0.9912865	Species
Bacteroides_vulgatus	IBS_vs_HC	0.14845835	0.8980479	0.9912865	Species
Hafnia_alveii	IBS_vs_HC	-0.1460735	0.88039282	0.9912865	Species
Clostridium_difficile	IBS_vs_HC	-0.2632568	0.7639382	0.9912865	Species
Clostridium_scindens	IBS_vs_HC	0.86643547	0.4335916	0.99106651	Species
Fusobacterium_nucleatum	IBS_vs_HC	1.00774193	0.04700261	0.37602085	Species
Oxalobacter_formigenes	IBS_vs_HC	-0.2818693	0.72317927	0.9912865	Species
Anaerotruncus_colihominis	IBS_vs_HC	-0.0984663	0.85930799	0.9912865	Species
Saccharomyces_cerevisiae	IBS_vs_HC	1.55986785	0.15074114	0.53801561	Species
Akkermansia_muciniphila	AI_vs_AG	-2.0018787	0.24812624	0.5671457	Species
Prevotella_copri	AI_vs_AG	6.8123945	0.00642544	0.1028071	Species
Faecalibacterium_prausnitzi i	AI_vs_AG	1.50318724	0.2012917	0.55162779	Species
Bilophila_wadsworthia	AI_vs_AG	0.32717578	0.74637736	0.79613585	Species
Bifidobacterium_adolescentis	AI_vs_AG	3.35245643	0.01573393	0.12587141	Species
Bifidobacterium_dentium	AI_vs_AG	-0.4462686	0.68414711	0.78188241	Species
Lactobacillus_plantarum	AI_vs_AG	0.30309182	0.52473264	0.69964353	Species
Butyrivibrio_crossotus	AI_vs_AG	0.82774371	0.44786933	0.6514463	Species
Bacteroides_vulgatus	AI_vs_AG	0.53324843	0.65139968	0.78188241	Species
Hafnia_alveii	AI_vs_AG	-1.7310994	0.08391276	0.33565105	Species
Clostridium_difficile	AI_vs_AG	1.86866693	0.03987447	0.21266385	Species
Clostridium_scindens	AI_vs_AG	-1.4278126	0.20686042	0.55162779	Species
Fusobacterium_nucleatum	AI_vs_AG	-0.4104794	0.4195861	0.6514463	Species
Oxalobacter_formigenes	AI_vs_AG	-0.7042428	0.38589603	0.6514463	Species
Anaerotruncus_colihominis	AI_vs_AG	-0.5920411	0.29734106	0.59468212	Species
Saccharomyces_cerevisiae	AI_vs_AG	0.22026391	0.8405174	0.8405174	Species

Akkermansia_muciniphila	HC_vs_AG	-2.320912	0.22252158	0.9471177	Species
Prevotella_copri	HC_vs_AG	0.53628166	0.83960776	0.965182	Species
Faecalibacterium_prausnitzii	HC_vs_AG	0.91998869	0.47355885	0.9471177	Species
Bilophila_wadsworthia	HC_vs_AG	0.06150778	0.95577381	0.965182	Species
Bifidobacterium_adolescentis	HC_vs_AG	3.0264491	0.04501938	0.360155	Species
Bifidobacterium_dentium	HC_vs_AG	0.15501429	0.89742373	0.965182	Species
Lactobacillus_plantarum	HC_vs_AG	-0.099826	0.84827858	0.965182	Species
Butyrivibrio_crossotus	HC_vs_AG	-1.0758553	0.36897454	0.9471177	Species
Bacteroides_vulgatus	HC_vs_AG	1.15603028	0.37315224	0.9471177	Species
Hafnia_alveii	HC_vs_AG	0.67684013	0.53293985	0.94744862	Species
Clostridium_difficile	HC_vs_AG	2.06698636	0.03829054	0.360155	Species
Clostridium_scindens	HC_vs_AG	-1.3371821	0.28021153	0.9471177	Species
Fusobacterium_nucleatum	HC_vs_AG	-0.4075184	0.46481495	0.9471177	Species
Oxalobacter_formigenes	HC_vs_AG	-0.1909822	0.82971215	0.965182	Species
Anaerotruncus_colihominis	HC_vs_AG	0.0270656	0.965182	0.965182	Species
Saccharomyces_cerevisiae	HC_vs_AG	-0.1143521	0.92410943	0.965182	Species
Akkermansia_muciniphila	IBS_vs_AG	-0.431404	0.76131027	0.81206429	Species
Prevotella_copri	IBS_vs_AG	2.52830729	0.20725764	0.47373175	Species
Faecalibacterium_prausnitzii	IBS_vs_AG	0.09238748	0.92349988	0.92349988	Species
Bilophila_wadsworthia	IBS_vs_AG	0.60268242	0.47053013	0.75999552	Species
Bifidobacterium_adolescentis	IBS_vs_AG	1.62094798	0.14932185	0.47373175	Species
Bifidobacterium_dentium	IBS_vs_AG	-0.625166	0.48981121	0.75999552	Species
Lactobacillus_plantarum	IBS_vs_AG	0.22296592	0.56999664	0.75999552	Species
Butyrivibrio_crossotus	IBS_vs_AG	-1.4227065	0.11655304	0.47373175	Species
Bacteroides_vulgatus	IBS_vs_AG	0.46435897	0.63306186	0.77915306	Species
Hafnia_alveii	IBS_vs_AG	-0.3093631	0.70410406	0.80469035	Species
Clostridium_difficile	IBS_vs_AG	0.96359987	0.19320885	0.47373175	Species
Clostridium_scindens	IBS_vs_AG	-1.3108763	0.16034475	0.47373175	Species
Fusobacterium_nucleatum	IBS_vs_AG	-0.2399061	0.5664231	0.75999552	Species
Oxalobacter_formigenes	IBS_vs_AG	-1.3129811	0.05299832	0.43498243	Species
Anaerotruncus_colihominis	IBS_vs_AG	-0.9115304	0.0543728	0.43498243	Species
Saccharomyces_cerevisiae	IBS_vs_AG	0.60538613	0.502898	0.75999552	Species
Akkermansia_muciniphila	AG_vs_AI	2.00187873	0.24812624	0.5671457	Species
Prevotella_copri	AG_vs_AI	-6.8123945	0.00642544	0.1028071	Species
Faecalibacterium_prausnitzii	AG_vs_AI	-1.5031872	0.2012917	0.55162779	Species
Bilophila_wadsworthia	AG_vs_AI	-0.3271758	0.74637736	0.79613585	Species
Bifidobacterium_adolescentis	AG_vs_AI	-3.3524564	0.01573393	0.12587141	Species
Bifidobacterium_dentium	AG_vs_AI	0.44626864	0.68414711	0.78188241	Species

Lactobacillus_plantarum	AG_vs_AI	-0.3030918	0.52473264	0.69964353	Species
Butyrivibrio_crossotus	AG_vs_AI	-0.8277437	0.44786933	0.6514463	Species
Bacteroides_vulgatus	AG_vs_AI	-0.5332484	0.65139968	0.78188241	Species
Hafnia_alveii	AG_vs_AI	1.73109944	0.08391276	0.33565105	Species
Clostridium_difficile	AG_vs_AI	-1.8686669	0.03987447	0.21266385	Species
Clostridium_scindens	AG_vs_AI	1.42781256	0.20686042	0.55162779	Species
Fusobacterium_nucleatum	AG_vs_AI	0.41047943	0.4195861	0.6514463	Species
Oxalobacter_formigenes	AG_vs_AI	0.70424275	0.38589603	0.6514463	Species
Anaerotruncus_colihominis	AG_vs_AI	0.59204109	0.29734106	0.59468212	Species
Saccharomyces_cerevisiae	AG_vs_AI	-0.2202639	0.8405174	0.8405174	Species
Akkermansia_muciniphila	HC_vs_AI	-0.1211293	0.95057174	0.95288327	Species
Prevotella_copri	HC_vs_AI	-6.0782088	0.02992385	0.23939079	Species
Faecalibacterium_prausnitzi i	HC_vs_AI	-0.3852946	0.77117075	0.95288327	Species
Bilophila_wadsworthia	HC_vs_AI	-0.067764	0.95288327	0.95288327	Species
Bifidobacterium_adolescentis	HC_vs_AI	-0.1281033	0.93338897	0.95288327	Species
Bifidobacterium_dentium	HC_vs_AI	0.79918693	0.52114059	0.95288327	Species
Lactobacillus_plantarum	HC_vs_AI	-0.2050139	0.70410852	0.95288327	Species
Butyrivibrio_crossotus	HC_vs_AI	-1.705695	0.17047817	0.82351873	Species
Bacteroides_vulgatus	HC_vs_AI	0.82068586	0.54014467	0.95288327	Species
Hafnia_alveii	HC_vs_AI	2.60584358	0.0231496	0.23939079	Species
Clostridium_difficile	HC_vs_AI	0.39622344	0.69546132	0.95288327	Species
Clostridium_scindens	HC_vs_AI	0.28853447	0.82082108	0.95288327	Species
Fusobacterium_nucleatum	HC_vs_AI	0.20086507	0.72703317	0.95288327	Species
Oxalobacter_formigenes	HC_vs_AI	0.71116456	0.43979573	0.95288327	Species
Anaerotruncus_colihominis	HC_vs_AI	0.81701069	0.20587968	0.82351873	Species
Saccharomyces_cerevisiae	HC_vs_AI	-0.136712	0.91230376	0.95288327	Species
Akkermansia_muciniphila	IBS_vs_AI	1.61192969	0.28640333	0.65463619	Species
Prevotella_copri	IBS_vs_AI	-4.2426323	0.04815265	0.38522119	Species
Faecalibacterium_prausnitzi i	IBS_vs_AI	-1.3693448	0.18273399	0.58474878	Species
Bilophila_wadsworthia	IBS_vs_AI	0.31696155	0.71973511	0.95964681	Species
Bifidobacterium_adolescentis	IBS_vs_AI	-1.6900535	0.15581708	0.58474878	Species
Bifidobacterium_dentium	IBS_vs_AI	-0.1374425	0.88582297	0.97874067	Species
Lactobacillus_plantarum	IBS_vs_AI	-0.038671	0.92581113	0.97874067	Species
Butyrivibrio_crossotus	IBS_vs_AI	-2.2089953	0.023135	0.37016006	Species
Bacteroides_vulgatus	IBS_vs_AI	-0.0274345	0.97874067	0.97874067	Species
Hafnia_alveii	IBS_vs_AI	1.4631913	0.09400603	0.50136551	Species
Clostridium_difficile	IBS_vs_AI	-0.8636121	0.27004162	0.65463619	Species
Clostridium_scindens	IBS_vs_AI	0.15839119	0.87167617	0.97874067	Species
Fusobacterium_nucleatum	IBS_vs_AI	0.21202825	0.63234557	0.95356953	Species

Oxalobacter_formigenes	IBS_vs_AI	-0.5672835	0.423503	0.847006	Species
Anaerotruncus_colihominis	IBS_vs_AI	-0.2780344	0.57376351	0.95356953	Species
Saccharomyces_cerevisiae	IBS_vs_AI	0.42657713	0.65557905	0.95356953	Species
Akkermansia_muciniphila	AG_vs_IBS	0.43140396	0.76131027	0.81206429	Species
Prevotella_copri	AG_vs_IBS	-2.5283073	0.20725764	0.47373175	Species
Faecalibacterium_prausnitzii	AG_vs_IBS	-0.0923875	0.92349988	0.92349988	Species
Bilophila_wadsworthia	AG_vs_IBS	-0.6026824	0.47053013	0.75999552	Species
Bifidobacterium_adolescentis	AG_vs_IBS	-1.620948	0.14932185	0.47373175	Species
Bifidobacterium_dentium	AG_vs_IBS	0.62516601	0.48981121	0.75999552	Species
Lactobacillus_plantarum	AG_vs_IBS	-0.2229659	0.56999664	0.75999552	Species
Butyrivibrio_crossotus	AG_vs_IBS	1.42270653	0.11655304	0.47373175	Species
Bacteroides_vulgatus	AG_vs_IBS	-0.464359	0.63306186	0.77915306	Species
Hafnia_alveii	AG_vs_IBS	0.30936306	0.70410406	0.80469035	Species
Clostridium_difficile	AG_vs_IBS	-0.9635999	0.19320885	0.47373175	Species
Clostridium_scindens	AG_vs_IBS	1.31087629	0.16034475	0.47373175	Species
Fusobacterium_nucleatum	AG_vs_IBS	0.23990609	0.5664231	0.75999552	Species
Oxalobacter_formigenes	AG_vs_IBS	1.31298114	0.05299832	0.43498243	Species
Anaerotruncus_colihominis	AG_vs_IBS	0.91153038	0.0543728	0.43498243	Species
Saccharomyces_cerevisiae	AG_vs_IBS	-0.6053861	0.502898	0.75999552	Species
Akkermansia_muciniphila	AI_vs_IBS	-1.6119297	0.28640333	0.65463619	Species
Prevotella_copri	AI_vs_IBS	4.2426323	0.04815265	0.38522119	Species
Faecalibacterium_prausnitzii	AI_vs_IBS	1.36934484	0.18273399	0.58474878	Species
Bilophila_wadsworthia	AI_vs_IBS	-0.3169615	0.71973511	0.95964681	Species
Bifidobacterium_adolescentis	AI_vs_IBS	1.69005354	0.15581708	0.58474878	Species
Bifidobacterium_dentium	AI_vs_IBS	0.13744246	0.88582297	0.97874067	Species
Lactobacillus_plantarum	AI_vs_IBS	0.03867098	0.92581113	0.97874067	Species
Butyrivibrio_crossotus	AI_vs_IBS	2.20899532	0.023135	0.37016006	Species
Bacteroides_vulgatus	AI_vs_IBS	0.02743454	0.97874067	0.97874067	Species
Hafnia_alveii	AI_vs_IBS	-1.4631913	0.09400603	0.50136551	Species
Clostridium_difficile	AI_vs_IBS	0.86361215	0.27004162	0.65463619	Species
Clostridium_scindens	AI_vs_IBS	-0.1583912	0.87167617	0.97874067	Species
Fusobacterium_nucleatum	AI_vs_IBS	-0.2120283	0.63234557	0.95356953	Species
Oxalobacter_formigenes	AI_vs_IBS	0.56728347	0.423503	0.847006	Species
Anaerotruncus_colihominis	AI_vs_IBS	0.27803437	0.57376351	0.95356953	Species
Saccharomyces_cerevisiae	AI_vs_IBS	-0.4265771	0.65557905	0.95356953	Species
Akkermansia_muciniphila	HC_vs_IBS	-2.7296377	0.11098137	0.53801561	Species
Prevotella_copri	HC_vs_IBS	-2.8321553	0.2357253	0.6286008	Species
Faecalibacterium_prausnitzii	HC_vs_IBS	-0.0125285	0.9912865	0.9912865	Species

Bilophila_wadsworthia	HC_vs_IBS	-1.3813043	0.16812988	0.53801561	Species
Bifidobacterium_adolescentis	HC_vs_IBS	0.56537146	0.67049547	0.9912865	Species
Bifidobacterium_dentium	HC_vs_IBS	-0.0599494	0.95561361	0.9912865	Species
Lactobacillus_plantarum	HC_vs_IBS	-1.1629216	0.01544231	0.24707698	Species
Butyrivibrio_crossotus	HC_vs_IBS	-0.4932784	0.64474127	0.9912865	Species
Bacteroides_vulgatus	HC_vs_IBS	-0.1484584	0.8980479	0.9912865	Species
Hafnia_alveii	HC_vs_IBS	0.14607353	0.88039282	0.9912865	Species
Clostridium_difficile	HC_vs_IBS	0.26325683	0.7639382	0.9912865	Species
Clostridium_scindens	HC_vs_IBS	-0.8664355	0.4335916	0.99106651	Species
Fusobacterium_nucleatum	HC_vs_IBS	-1.0077419	0.04700261	0.37602085	Species
Oxalobacter_formigenes	HC_vs_IBS	0.28186928	0.72317927	0.9912865	Species
Anaerotruncus_colihominis	HC_vs_IBS	0.09846631	0.85930799	0.9912865	Species
Saccharomyces_cerevisiae	HC_vs_IBS	-1.5598679	0.15074114	0.53801561	Species

Table S2. Spearman correlation analysis between gut microbiota relative abundances and clinical parameters, including short-chain fatty acids and metabolic markers. Correlation analysis was performed using Spearman's rank correlation coefficient (ϱ). Correlations were calculated between microbial relative abundances and clinical parameters, including short-chain fatty acids (acetate, propionate, butyrate, lactate), body mass index (BMI), lipid profile (total cholesterol, LDL, HDL), and vitamin D levels. P-values were adjusted for multiple testing using the false discovery rate (FDR) method. Statistically significant correlations (FDR-adjusted p < 0.05) are indicated in bold.

Microbiome	Clinical	n	Spearman_r	p_value	p_adj_FDR
Actinobacteria	Lactate	59	0.91	0	0
Actinobacteria	Butyrate	59	0.001	0.9923	0.9923
Actinobacteria	Acetate.Propionate	59	0.188	0.1547	0.4368
Actinobacteria	BMI	59	-0.13	0.3262	0.6929
Actinobacteria	Vitamin.D	59	0.022	0.8663	0.9537
Actinobacteria	Total.Cholesterol	59	-0.383	0.0028	0.0149
Actinobacteria	LDL	59	-0.21	0.1102	0.3555
Actinobacteria	HDL	59	-0.067	0.6141	0.9211
Bifidobacterium	Lactate	59	0.941	0	0
Bifidobacterium	Butyrate	59	0.018	0.8898	0.9537
Bifidobacterium	Acetate.Propionate	59	0.267	0.0407	0.1785
Bifidobacterium	BMI	59	-0.181	0.1709	0.4557
Bifidobacterium	Vitamin.D	59	0.011	0.9338	0.9537

Bifidobacterium	Total.Cholesterol	59	-0.409	0.0013	0.0078
Bifidobacterium	LDL	59	-0.215	0.1024	0.3555
Bifidobacterium	HDL	59	-0.102	0.443	0.7694
Bacteroides	Lactate	59	0.122	0.356	0.6929
Bacteroides	Butyrate	59	-0.149	0.2597	0.5936
Bacteroides	Acetate.Propionate	59	0.955	0	0
Bacteroides	BMI	59	-0.034	0.7986	0.9537
Bacteroides	Vitamin.D	59	0.128	0.3337	0.6929
Bacteroides	Total.Cholesterol	59	-0.046	0.729	0.9296
Bacteroides	LDL	59	0.013	0.9216	0.9537
Bacteroides	HDL	59	-0.056	0.671	0.9296
Firmicutes	Lactate	59	-0.052	0.6955	0.9296
Firmicutes	Butyrate	59	0.576	0	0
Firmicutes	Acetate.Propionate	59	-0.51	0	0
Firmicutes	BMI	59	0.072	0.5883	0.9211
Firmicutes	Vitamin.D	59	-0.09	0.4989	0.8258
Firmicutes	Total.Cholesterol	59	0.059	0.6575	0.9296
Firmicutes	LDL	59	0.101	0.4488	0.7694
Firmicutes	HDL	59	0.021	0.8724	0.9537
Bacteroidetes	Lactate	59	-0.121	0.3609	0.6929
Bacteroidetes	Butyrate	59	-0.267	0.0409	0.1785
Bacteroidetes	Acetate.Propionate	59	0.548	0	0
Bacteroidetes	BMI	59	0.167	0.2075	0.498
Bacteroidetes	Vitamin.D	59	-0.115	0.3854	0.7115
Bacteroidetes	Total.Cholesterol	59	-0.014	0.9141	0.9537
Bacteroidetes	LDL	59	0.041	0.7603	0.9358
Bacteroidetes	HDL	59	-0.21	0.1111	0.3555
Prevotella	Lactate	59	-0.215	0.1016	0.3555
Prevotella	Butyrate	59	0.024	0.8577	0.9537
Prevotella	Acetate.Propionate	59	-0.644	0	0
Prevotella	BMI	59	0.172	0.192	0.4851
Prevotella	Vitamin.D	59	-0.205	0.1186	0.3558
Prevotella	Total.Cholesterol	59	0.045	0.7359	0.9296
Prevotella	LDL	59	-0.068	0.6074	0.9211
Prevotella	HDL	59	-0.046	0.7294	0.9296
